# An evolutionary look into the history of lentil reveals unexpected diversity

**DOI:** 10.1111/eva.13467

**Published:** 2022-08-21

**Authors:** Azalea Guerra‐Garcia, Teketel Haile, Ezgi Ogutcen, Kirstin E. Bett, Eric J. von Wettberg

**Affiliations:** ^1^ Department of Plant Sciences University of Saskatchewan Saskatoon Saskatchewan Canada; ^2^ Conservatoire et Jardin Botaniques de la Ville de Genève Geneva Switzerland; ^3^ Plant and Soil Science and Gund Institute for the Environment University of Vermont Burlington Vermont USA

**Keywords:** copy number variation, crop diversity, crop domestication, exome capture, legumes, lentils

## Abstract

The characterization and preservation of genetic variation in crops is critical to meeting the challenges of breeding in the face of changing climates and markets. In recent years, the use of single nucleotide polymorphisms (SNPs) has become routine, allowing us to understand the population structure, find divergent lines for crosses, and illuminate the origin of crops. However, the focus on SNPs overlooks other forms of variation, such as copy number variation (CNVs). Lentil is the third most important cold‐season legume and was domesticated in the Fertile Crescent. We genotyped 324 accessions that represent its global diversity, and using both SNPs and CNVs, we dissected the population structure and genetic variation, and identified candidate genes. Eight clusters were detected, most of them located in or near the Fertile Crescent, even though different clusters were present in distinct regions. The cluster from South Asia was particularly differentiated and presented low diversity, contrasting with the clusters from the Mediterranean and the northern temperate. Accessions from North America were mainly assigned to one cluster and were highly diverse, reflecting the efforts of breeding programs to integrate variation. Thirty‐three genes were identified as candidates under selection and among their functions were sporopollenin synthesis in pollen, a component of chlorophyll B reductase that partially determines the antenna size, and two genes related to the import system of chloroplasts. Eleven percent of all lentil genes and 21% of lentil disease resistance genes were affected by CNVs. The gene categories overrepresented in these genes were “enzymes,” “Cell Wall Organization,” and “external stimuli response.” All the genes found in the latter were associated with pathogen response. CNVs provided information about population structure and might have played a role in adaptation. The incorporation of CNVs in diversity studies is needed for a broader understanding of how they evolve and contribute to domestication.

## 1 INTRODUCTION

Crop diversity is the result of past and current natural and human‐mediated evolutionary processes, which continue as farmers and breeders select traits of interest. Understanding the distribution of genetic variation in crops allows us to elucidate these evolutionary processes and is critical to our efforts to preserve and harness that variation, which is crucial to meet the challenges of breeding in the face of rapidly changing markets, climates, and agricultural approaches. Landraces also play a role in food security in regions where traditional and small‐scale agriculture occurs, and are a unique source of variation (Camacho‐Villa et al., 2005; Mercer & Perales, 2010). Domestication, historical human migrations, and different culinary traditions, coupled with mutation, natural selection, and breeding, have all crafted crop genetic variation. Often these processes have distinct effects since some reduce the diversity, meanwhile others mix, isolate or promote variation. Regional variation is observed in many adaptive traits such as abiotic stress tolerance, flowering time, and disease resistance, and selection based on these factors further improves the domesticated crops, enabling them to adapt to a variety of local environmental conditions (Hoban et al., 2016). In many crops, genetic diversity has been eroded over time due to small effective population sizes and repeated intercrossing, selection, and propagation of uniform and stable cultivars (e.g., Gross, 2012; Morrell et al., 2012; Warschefsky et al., 2014). The loss of genetic diversity is further aggravated by the increased demand for homogeneous monocropping and, due to climate change and habitat loss of the wild relatives, the reduction in genetic diversity is becoming an acute threat to many crop species.

In contrast to the routine use of Single Nucleotide Polymorphisms (SNPs) to examine the evolutionary history of crops and to identify causal variants, the role of Copy Number Variation (CNV) in the domestication process had been overlooked until recently (Gaut et al., 2018). CNVs are polymorphisms that differ in the number of copies of a specific sequence present between individuals of one species and include deletions, duplications, and insertions of >1 kb size Freeman et al., 2006. CNVs are likely to have large effects on phenotypes, which make them more likely to be deleterious (Emerson et al., 2008; Katju & Bergthorsson, 2013; Zhang et al., 2009). Despite this, CNVs might actually be a source of diversity, facilitating adaptation since the few CNVs retained could have an important role in the phenotypic variation (Lye & Purugganan, 2019). Therefore, significant variation might not be captured in solely SNP‐based studies Bai et al., 2016. CNVs have been characterized in some major crops, such as maize (Sun et al., 2018; Swanson‐Wagner et al., 2010), potato (Hardigan et al., 2016; Knaus et al., 2020), tomato (Alonge et al., 2020) and grapevine (Mercenaro et al., 2017; Zhou et al., 2019), but population genetic studies based on CNVs in domesticated crops are still scarce.

Due to their association with nitrogen‐fixing bacteria, legume crops are an essential part of many agricultural systems. Introducing legumes into crop rotations helps to replenish the nitrogen in the soil, thereby reducing the use of chemical fertilizers and promoting environmentally friendly agricultural systems. Lentil (*Lens culinaris* Medik.) is an annual self‐pollinating legume, and one of the oldest crops with a domestication history dating back to 11,000 BP in the Fertile Crescent (Ladizinsky, 1979). Lentil is the third most important cool season grain legume and is a source of food in many cultures. It is currently cultivated in about 45 countries, concentrated in regions such as temperate North America, the Mediterranean coasts, the Middle East, South Asia, and East Africa (Food and Agriculture Organization of the United Nations Statistical Database; FAOSTAT, 2020).

Lentil is a diploid (2*n* = 14) organism, but the complexity of its large genome (4063 Mbp; Arumuganathan & Earle, 1991) makes this important crop difficult to study. An early study demonstrated low genetic diversity in cultivated lentils due to bottlenecks associated with lentil spread in South Asia Erskine et al., 1998. Later, more tools and resources became available for lentils and several studies addressing its population structure and genetic diversity have been conducted (Dissanayake et al., 2020; Ferguson et al., 1998; Idrissi et al., 2015; Khazaei et al., 2016; Liber et al., 2021; Lombardi et al., 2014; Pavan et al., 2019). Most of these studies are based on low‐resolution markers, including a relatively low number of loci, and/or are focused on accessions from a particular region. We recently developed an exome capture array for lentils (Ogutcen et al., 2018) and generated high‐quality genomic data from a lentil panel integrated by accessions that represent the global lentil diversity.

In this study, using both SNPs and CNVs we aim to (i) dissect the population structure within and between cultivated lentil populations from around the world, (ii) examine the effects of domestication on the genetic diversity of lentils, and (iii) identify the genomic basis of adaptation in this legume. This work will provide a foundation for candidate gene search for agronomically important traits and can be essential to enrich the cultivated lentil germplasm and to further improve lentil breeding programs.

## 2 MATERIAL AND METHODS

### 2.1 Lentil diversity panel and exome capture

We genotyped a lentil diversity panel (LDP), consisting of 324 lentil accessions that represented the global diversity of cultivated lentils (Haile et al., 2020; Wright et al., 2021). This panel contained samples from all of the major areas of lentil domestication and postdomestication divergence (Table S1). The genotypes used were derived from a combination of landraces and bred varieties gathered from genebanks of the United States Department of Agriculture (USDA), the International Center for Agricultural Research in the Dry Areas (ICARDA), Plant Gene Resources of Canada (PGRC), and the Crop Development Center of the University of Saskatchewan, Canada. The exome capture assay developed by Ogutcen et al. (2018) was used to genotype one plant per accession of the LDP.

### 2.2 SNP calling and filtering

An initial quality filter was applied to the raw reads with Trimmomatic 0.36 (Bolger et al., 2014) using the following parameters: maximum N content error of 10%; minimum base median quality PHRED score = 28; minimum per‐sequence quality = 25; minimum quality in a four‐base window = 30; and reads longer than 50 bp. The alignment of the trimmed reads to the reference genome (Ramsay et al., 2021) was performed with Bowtie2 2.3.3.1 (Langmead & Salzberg, 2012). In order to remove PCR duplicates, we used the rmdup function from SAMtools 1.3.1 (Li et al., 2009). Variants were called using SAMtools, setting a minimum number of gapped reads to call an indel to 10. After variant discovery, SNPs were filtered with VCFtools 0.1.15 (Danecek et al., 2011) using a minimum quality = 30, minimum mean depth = 3×, maximum missingness per site = 0.10, minimum allele count (MAC) = 5, the SNPs within the CNV were removed (see Section 3), and only biallelic loci were kept.

### 2.3 CNV discovery and characterization

Alignment files were merged using Sambamba (Tarasov et al., 2015) and FAI index files were created with Samtools (Li et al., 2009). The depth of sequencing coverage was then estimated with the DepthOfCoverage tool from the Genome Analysis Toolkit (GATK 4.1.8.1; McKenna et al., 2010). The discovery and filtering of CNVs were performed with the Exome Hidden Markov Model (XHMM; McKenna et al., 2010) using a minimum target size of 100 bp, a minimum mean target read depth of 3×, and a maximum of 3000×. XHMM uses principal component analysis (PCA) to normalize exome read depth and a hidden Markov model (HMM) to discover the CNV and genotype variation across samples.

In order to test whether there was an association between the number of CNV per chromosome and the chromosome size or the number of genes within them, Spearman correlation tests were performed.

### 2.4 Population structure and genetic diversity

Population structure and genetic diversity were assessed using both SNPs and CNVs. Clusters were inferred with the SNPs using the “simple” prior in fastSTRUCTURE, a variational Bayesian framework for posterior inference (Raj et al., 2014). To detect the optimal number of subpopulations (K), we ran a series of analyses with increasing K from one to ten with five cross‐validation runs for each K. The range of optimal K was determined using the “chooseK” function in fastSTRUCTURE. The population structure of lentils was also explored through PCA. In the case of the SNPs, the PCA was performed with SNPrelate (Zheng et al., 2012). For the CNVs, the prcomp R function (R Core Team, 2020) was used, applying the analysis to the normalized read depth of the loci identified as deletions or duplications.

After CNV discovery, the number of deletions and duplications per accession within each cluster and their length were estimated. Kruskal‐Wallis and Wilcox tests with the Holm correction were applied to compare those attributes among clusters. An R script that estimated the frequency of CNVs within each population was used, and then a custom Rscript was used to estimate the frequency of CNVs and the identification of private CNVs based on the established populations. The functional classification of the genes found within the CNVs was performed through MapMan4 (Schwacke et al., 2019) and a *χ* ^2^ test computing p‐values by Monte Carlo simulation (10,000 simulations) was performed in order to test whether the affected genes were randomly distributed among the gene categories Only genes that were present at a frequency higher than 0.20 in each cluster were classified.

Pairwise *F*_ST_ (Weir & Cockerham, 1984) comparisons were performed with the SNPs using a sliding window approach in VCFtools v0.1.15 (Danecek et al., 2011). For each comparison, the mean *F*_ST_ value was calculated in 100 kb sliding windows with a step size of 50 kb. To estimate genetic differentiation based on the CNVs, we calculated the variant fixation index (*V*_ST_), which is an analog of *F*_ST_ that has been used to evaluate the divergence between populations from CNV loci (Dorant et al., 2020; Redon et al., 2006; Rinker et al., 2019). For each pairwise population comparison, *V*_ST_ = (*V*_T_ − *V*_S_)/*V*_T_, where *V*_T_ is the variance of the normalized read depths of all individuals from the two populations, and *V*_S_ is the weighted mean of the variance within each population (Redon et al., 2006).

Tajima's D statistic (Tajima, 1989) was calculated from biallelic SNPs in 100 kb nonoverlapping windows across the genome of all individuals in each structure cluster using VCFtools. Since the number of polymorphic sites and private alleles is dependent on the sample size and the number of accessions per cluster differs, a rarefaction approach was applied for allelic richness and private allelic richness using ADZE v1.0 (Szpiech et al., 2008).

The Site Frequency Spectrum (SFS) of each cluster was constructed with both SNPs and CNVs. The allele count function of VCFtools was used for the SNP data and a custom Rscript for the count of CNVs. The expected SFS was derived using the Watterson estimator ${\hat{\theta }}_{W}=\frac{s}{\sum_{i=1}^{n-1}\frac{1}{i}}$, where *i* is the number of samples and *n* is the number of SNPs. The two clusters distributed on the Mediterranean coast are also distributed in America (see Section 3). For these clusters, the rarefaction diversity analysis was performed subdividing according to whether they were from the Americas or the Mediterranean region.

### 2.5 Selection tests

Three methods were applied to identify regions under selection. The first method, SelectionHapStats (https://github.com/ngarud/SelectionHapStats), looks for selective sweeps in the genome by calculating haplotype homozygosity statistics (H12) to identify genomic regions that have undergone recent and strong adaptation. We scanned the genome using sliding windows of 50 SNPs with intervals of 5 SNPs between window centers and calculated H12 in each window. H12 peaks were identified using the “H12peakFinder” function in SelectionHapStats, keeping the top 5 peaks per chromosome longer than the average LD decay (150 kb) to avoid false positives.

It is expected that the selective sweeps in autogamous species will tend to be longer due to the reduction in effective recombination. To reduce the number of candidate genes under selection and the false‐positive rate, the other two selected methods are based on outlier detection: the R package pcadapt (Luu et al., 2017) and BayeScan 2.1 (Foll & Gaggiotti, 2008). For these outlier tests, we applied second filtering to the data set: minimum mean depth = 3×, minimum allele count = 10, only keeping biallelic variants and removing SNPs closer than 2000 bp and located within CNV regions. Pcadapt detects candidate loci under selection that are outliers with respect to the population structure inferred through a PCA. It does not require grouping samples and can handle admixed individuals, which is important when the clusters are not discrete units and present mixed ancestry (Luu et al., 2017). BayeScan uses differences in allele frequencies of predefined populations. In both outlier analyses, the significance threshold value was set to 0.01.

To avoid false positives, we only considered genes as “true candidates under selection” if they were detected by at least two of the three methods. The functional classification of these candidate genes under selection was performed using MapMan4 and a *χ* ^2^ test computing p‐value by Monte Carlo simulation (10,000 simulations) to check whether the candidate genes under selection were randomly distributed across the gene categories.

## 3 RESULTS

### 3.1 Genotyping

A total of 324 lentil accessions representing the global diversity were genotyped using an exome capture approach. After read mapping to a reference assembly, 34,637,608 SNPs were identified, of which 3,074,957 variants were kept after applying quality filters. The filtered data set presented a mean depth per site of 21.69× and a mean missing rate per site of 1.7%. The XHMM method detected a total of 2646 CNV regions affected across the lentil diversity panel.

### 3.2 Population structure and genetic diversity characterization using SNPs

**FIGURE 1:**
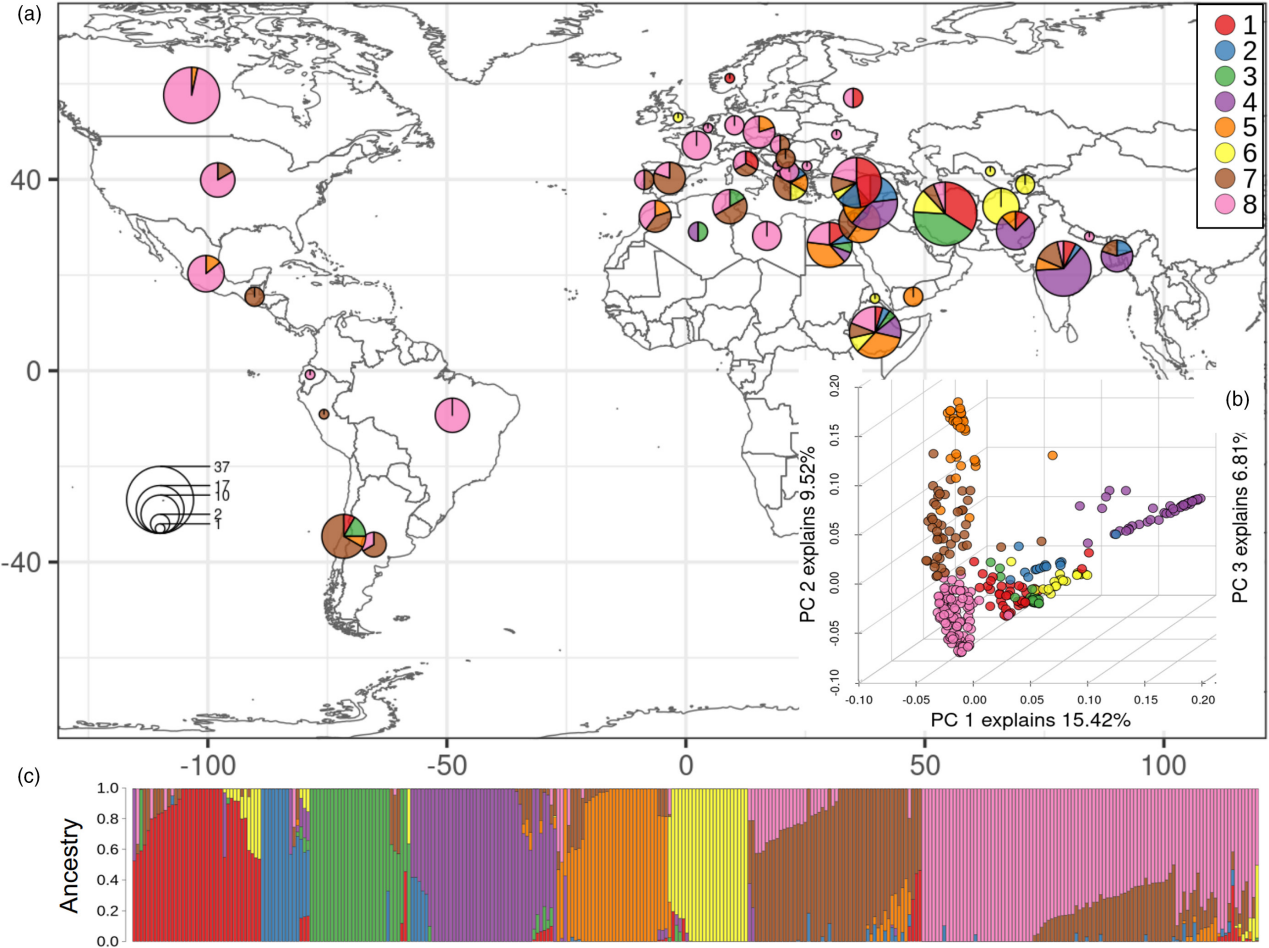
(a) Global distribution of the lentil diversity panel (LDP). The size of the circles represents the number of accessions from different countries, and the colors indicate the clusters to which they belong. (b) Principal component analysis (PCA) for the first three principal components using the single nucleotide polymorphism (SNP) data set. (c) Ancestry plot of the LDP. The distributions of the lentil genetic clusters are: 1 = the Middle East (Iran, Turkey); 2 = the Middle East (Syria, Turkey); 3 = Iran; 4 = South Asia (India, Pakistan) and Syria; 5 = East African highlands and South Levant (Ethiopia, Jordan, and Egypt); 6 = Central Asia (Afghanistan, Iran); 7 = Mediterranean costs (Spain) and South America (Chile); 8 = Temperate Mediterranean (France) and North America (Canada).

We found eight clusters with fastSTRUCTURE (Figure 1). K was estimated between one and ten, and we picked K = 8 as the best description based on the method described by Evanno et al. (2005). Even though most of the clusters presented mixed ancestry, there is a clear separation between the clusters found to the East and West of the lentil center of origin (Figure 1a). This pattern is also observed in the first principal component of the PCA (Figure 1b). Despite the fact that lentil clusters were not tightly defined geographically, there were distinct patterns in their distribution and some clusters are more common in particular regions (Table 1, Figure 1a, Figure S1). Several clusters were found within or close to the Middle East, which is the center of origin (Table 1, Figure S1), and the diversity in the number of clusters decreases with increasing distance from this region.

**TABLE 1:** Distribution of the lentil accessions included in this study

Population cluster	Distribution	*n*	*n* (>80% ancestry)
1	The Middle East (Iran, Turkey)	37	27
2	The Middle East (Syria, Turkey)	14	8
3	Iran	29	23
4	South Asia (India, Pakistan) and Syria	42	29
5	East African highlands and South Levant (Ethiopia, Jordan and Egypt)	32	21
6	Central Asia (Afghanistan, Iran)	23	22
7	Mediterranean costs (Spain); 7A South America (Chile)	50	23
8	Temperate Mediterranean (France); 8A North America (Canada)	97	51

*Note*: In clusters 7 and 8, indicates the main country in Europe and in the Americas, where lentil was more recently introduced.

The clusters that had the highest number of segregating sites were 1 (distributed in the Middle East), 8 (Mediterranean and North America; northern temperate climates) and 7 (Mediterranean coast and South America; Mediterranean climates), and 5 (East African highlands, South Levant and Egypt; Figure S2a). The highest genetic diversity in terms of allelic richness was found in clusters 7, 5, and 1 (Figure S3a). It is worth noting that the allelic richness of clusters 7 and 8 was relatively high among the accessions from the Mediterranean but dropped among the accessions distributed in the Americas (Figure S2a). This reduction in diversity is dramatic in the case of cluster 7A (mainly from South America), contrasting with the low loss of diversity in the accessions from cluster 8A distributed mostly in North America.

The clusters with the lowest diversity in terms of allelic richness and segregating sites were located in Central Asia South Asia and Iran (clusters 6, 4, and 3; Figure S3a). Cluster 2 also exhibited low levels of diversity; however, fewer accessions were included in this group in comparison with other clusters so this may not be truly representative of this cluster. The greatest private allele richness (Figure S3b) and the highest levels of differentiation (Figure S4) were found in cluster 5. Clusters 3, 4, and 6 also presented relatively large FST values compared with the rest of the populations (Figure S4). Conversely, the lowest differentiation levels were estimated in cluster 1.

**FIGURE 2:**
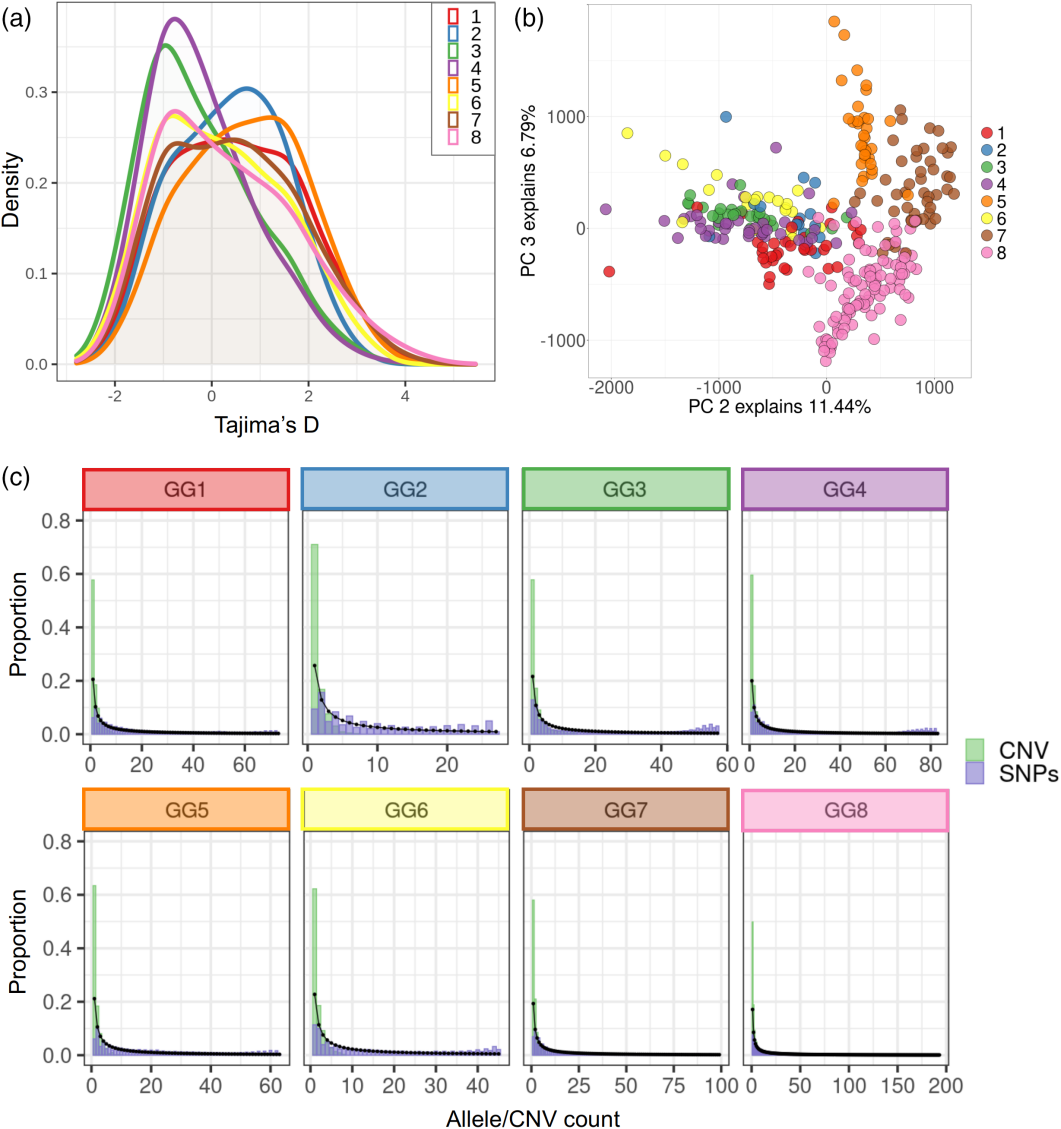
(a) Tajima's D distribution estimated for each of the eight population clusters estimated for the lentil diversity panel (LDP). (b) Principal component analysis (PCA) inferred from the normalized depth of copy number variation (CNV) loci with each population cluster represented by dots in different colors. (c) Site frequency spectrum (SFS) of the individual LDP clusters. The observed SFS constructed from single nucleotide polymorphisms (SNPs) and from CNVs is indicated with different bar colors. Black dots and lines show the expected SFS.

The genomic patterns of Tajima's D estimation varied across the clusters (Figure 2a). Cluster 8 (Temperate Mediterranean and North America), 6 (Central Asia), 3 (Iran), and 4 (Southeast Asia) had negative values; the last two having the lowest Tajima's D estimates. Positive peaks were found for clusters from the Middle East region (clusters 1 and 2), 5 (East African highlands), and a less conspicuous positive peak is observed in cluster 7 (Mediterranean region and South America).

A deficit of low‐frequency SNPs variants compared with the expected SFS was detected in all the clusters. Furthermore, an excess of high‐frequency alleles was observed in most of the populations (clusters 2, 3, 4, 5, and 6; Figure 2c).

### 3.3 CNV diversity in the LDP

Of the 2646 CNV regions identified, 1949 were duplications and 1827 were deletions. Some of the CNV regions were identified as duplications in some accessions and as deletions in other lentil samples. Therefore, the sum of the duplications and deletions did not correspond to the total number of CNV regions. On average, each genotype had 48 regions affected by a CNV ‐ both deletions and duplications (Figure S5a). The median lengths of deletions and duplications across all clusters were 21.87 kb and 25.96 kb, respectively (Figure S6). Clusters 1, 3, 6, 7, and 8 had longer deletions compared with the other clusters, but no differences in the length of duplications were found (Figure S6).

The count of CNVs within the clusters was highly dependent on the number of accessions and the cluster where we detected the most CNVs was cluster 8 (97 accessions), followed by cluster 7 (50 accessions; Figure S2b). Nevertheless, clusters 3 (24%) and 4 (23%) showed the highest proportions of private CNVs (Figure S5b).

The first principal component of the normalized read depth of CNVs explained 29.6% of the variance and no correspondence with the genetic clusters was observed (Figure S7a). Adding the second and third principal components, which explained the 11.4% and the 6.8% of variance, respectively, reflected a similar population structure to the one shown by the SNP data set (Figure 2b). The pattern observed in the pairwise *V*_ST_ was also congruent with the *F*_ST_ estimation, with clusters from the Middle East, South Asia, and East Africa being highly differentiated (Figure S7b). The highest differentiation was found in clusters 3, 5, and 7 (Figure S7b).

An excess of low‐frequency variants and a lack of high‐frequency ones were a constant pattern across all the SFS constructed with the CNV data set for all population clusters (Figure 2c).

**FIGURE 3:**
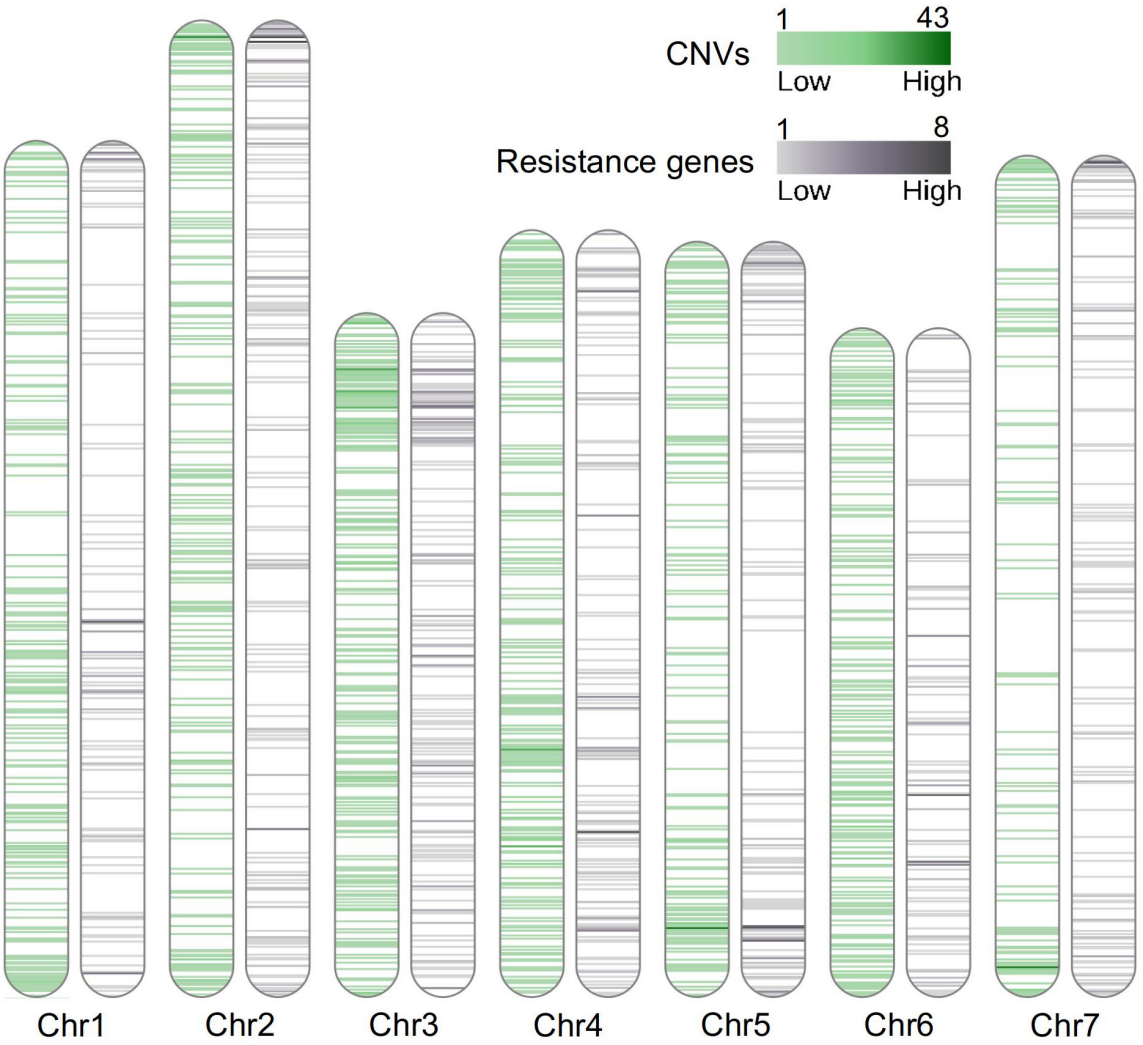
Distribution of copy number variation (CNV) loci along the lentil chromosomes. Green indicates the CNV density, and gray indicates the disease resistance gene frequency, in 1 mb windows.

There was no correlation between the chromosome size nor the number of genes in the chromosomes and the number of CNVs found (*p* = 0.24 and *p* = 0.84, Figure S8). Chromosome 3 contained the highest number of CNVs, despite being the second shortest chromosome. Furthermore, chromosome 2, which is the largest one and contains the greatest number of genes, did not contain a large number of CNVs (Figure 3 and Figure S8).

**FIGURE 4:**
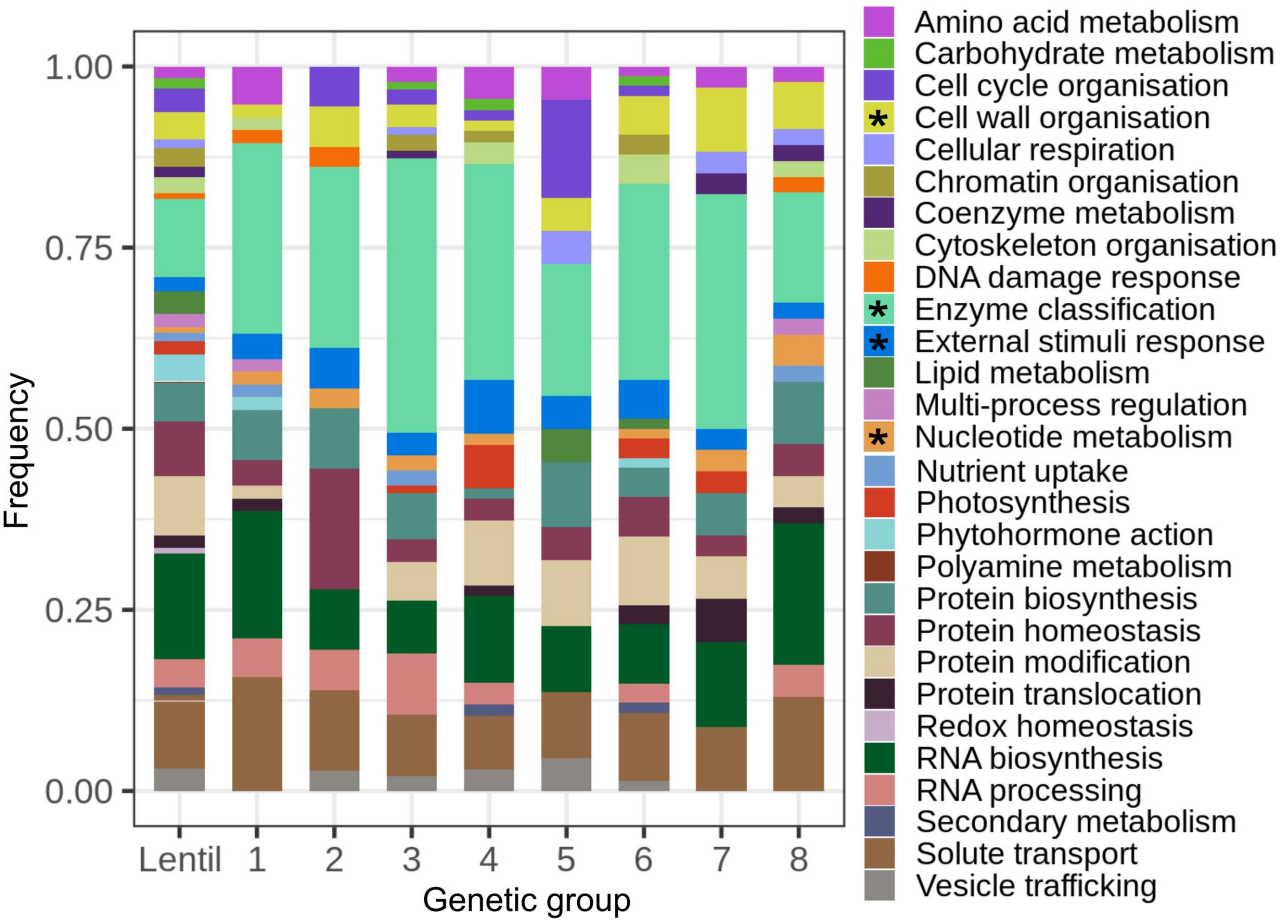
MapMan classification of the genes in copy number variation (CNV) regions. The first column shows the classification of all lentil genes, while the rest of the columns indicate the classification of genes affected by CNV loci that were presented within each cluster in a frequency higher than 0.20. The “Not assigned” category was not included in the figure. Asterisks show the categories that were overrepresented in the genes affected by CNVs. See Figure S9 for more details about subcategories of enzymes, nucleotide metabolism, external stimuli response, and Cell Wall Organization.

Of the ~58,200 genes predicted in lentils (Ramsay et al., 2021), 6722 (11.6%) were affected by CNVs. Using MapMan4, 5979 of these genes were classified into 29 categories, including a “Not assigned” one (Figure 4). Genes affected by CNVs were not randomly distributed among the MapMan4 gene categories (*p* < 0.001) and the top overrepresented categories were “enzymes,” “nucleotide metabolism,” “external stimuli response,” and “Cell Wall Organization.”

The most abundant category among the genes affected by CNVs was “Enzymes,” except in cluster 8, in which “RNA biosynthesis” was the most common (Figure 4). Within this “Enzymes” class, the transferase transferring phosphorus‐containing group was the most abundant but is also the most common among the enzymes present in lentil genes in general (Figure S9c). In the case of the “External stimuli response,” all the genes found in this category were related to pathogen response (Figure S8a), and in “Cell Wall Organization” the genes were mostly associated with the presence of xylan in the cell wall (Figure S9d).

Approximately 1150 (2.0%) of the lentil genes in the reference genome are predicted to be related to disease resistance (Ramsay et al., 2021) and 240 of them (20.8%) were associated with CNV regions (Figure 3), which is many more than expected by chance (*p* < 0.001).

### 3.4 Identifying regions under selection

Three methods were used to detect regions under selection in the lentil genome. Two of these are outlier approaches (pcadapt and BayeScan) and the third one is based on the detection of selective sweeps (SelectionHapStats). BayeScan detected 1312 SNPs located across 487 genes. Using the package pcadapt, the first five principal components were assessed since they best reflected the population structure and 799 SNPs, located across 485 genes, appear to be under selection. Finally, the top five selective sweeps per chromosome identified using SelectionHapStats included 516 genes.

In order to reduce false positives, only genes that were detected by at least two methods were considered true candidates under selection. Thirty‐three genes met this condition (Figure S10), which were then classified using MapMan into 14 categories (Figure S11) and no overrepresented category was found (*p* > 0.05). Protein modification was the category that contained the highest proportion of candidate genes under selection (Figure S11; Table S2). Three of the 33 candidate genes were not annotated. Among the annotated genes were a long‐chain fatty acid hydroxylase (Lcu.2RBY.7G006750, “Cell Wall Organization” category), which is involved in sporopollenin synthesis in pollen (Dobritsa et al., 2009); a component of chlorophyll B reductase (Lcu.2RBY.7G021070, “Coenzyme metabolism” category); protein TIC 22 (Lcu.2RBY.3G061880); and the component FtsH12 of a translocation ATPase motor complex in the chloroplast (Lcu.2RBY.7G021140). The last two genes were classified within the “Protein translocation” category (Table S2).

## 4 DISCUSSION

### 4.1 Genetic diversity and the geographic spread of lentil

We examined the genetic variation of lentils using both SNPs and CNV loci generated from an exome capture array in a lentil diversity panel, consisting of 324 accessions from 48 countries, representing global lentil diversity. Our results were consistent with most of the conclusions from previous work, and we were able to further detect population structure and to estimate diversity levels of accessions from different regions. Khazaei et al. (2016) identified three major clusters of cultivated lentil that reflected its domestication history and the agro‐ecological zones where this legume is produced: subtropical savannah, Mediterranean, and northern temperate. Our results from an expanded set of germplasm followed this general pattern and we identified further population structures resulting in eight clusters. Most of the clusters were located in or near the Fertile Crescent, the center of origin of lentils and from where lentils spread with the rise of agriculture. Many of the accessions had a mixed ancestry, and in the case of the highly diverse accessions from North America might reflect the breeding efforts over time. Mixed ancestry can also be due to gene flow and/or incomplete lineage sorting, which is likely in populations that diverged as recently as domesticated lentils. It is worth noting that lentil clusters were not discrete geographic units, but instead the predominant clusters change gradually throughout the geographic space. Different factors, both natural and human‐mediated, might have contributed to this, such as incomplete lineage sorting and gene flow due to natural factors and/or to the human seed exchange. The distribution patterns observed in the lentil clusters were: clusters 1 and 2 were mainly in the Middle East, cluster 3 in Iran, cluster 4 in South Asia, cluster 5 in Eastern Africa, cluster 6 in Central Asia, cluster 7 in the Mediterranean region and South America, and cluster 8 was mainly found in temperate climates around the Mediterranean and in North America.

Several genetic clusters were found in the Middle East and the genetic diversity in this area, particularly in cluster 1, was the highest in terms of the number of segregating sites and allele richness (Figures S2a and S3a). Cluster 2, which is also distributed in the Middle East, presented a relatively high number of segregating sites considering its low number of accessions (Figure S2a). By contrast, the clusters distributed mainly in South Asia (cluster 4), Iran (cluster 3), and Central Asia (cluster 6) had the lowest amount of variation, as was also observed by Khazaei et al. (2016), and were highly differentiated. Nonetheless, the cluster with the highest FST was from the East African highlands (cluster 5; Figures S4 and S7b). We found less differentiation among lentil clusters from the Middle East (clusters 1 and 2). This is expected since the latter clusters are distributed in the center of origin and probably represent older gene pools, whereas the other clusters resulted following the spread of lentils to new production areas and contain a subset of the variation found in this region.

Lentil is thought to have moved into South Asia from the Fertile Crescent with the arrival of Aryan people around ~4000–3500 years ago (Cubero et al., 2009) and several factors might have contributed to the decline in genetic variation such as bottlenecks associated with the spread, and nonoverlapping distributions with wild relatives (Ferguson et al., 1998), avoiding gene flow from wild lentil species. These factors might also be related to the low proportion of mixed ancestry observed in the cluster distributed in this area (cluster 4; Figure 1c). Non‐neutral evolutionary processes have probably also shaped the diversity, and strong selection in a novel climate zone could contribute to the decrease in variation. Photoperiod and temperature sensitivity have been suggested as key for lentil production (Summerfield et al., 1985; Wright et al., 2021), therefore it is likely that these environmental factors have played an important role in the variation of lentils.

Similar values of genetic variation to the clusters within or close to the center of origin of lentils were observed in the accessions from the Mediterranean region (clusters 7 and 8; Figure S3). A possible explanation for the maintenance of diversity in clusters 7 and 8 is a less severe bottleneck during the spread to these regions and/or several introductions from the center of origin due to intentional breeding efforts. Furthermore, sympatric distribution with the wild lentil species might have facilitated introgression from wild relatives around the Mediterranean. These clusters were also distributed in America (clusters 7A and 8A), but a reduction in diversity was observed in these accessions (Figure S3a).

Even though both clusters were distributed in the Mediterranean region, accessions from cluster 8 were mainly found in temperate regions, where the lentil production occurs during the summer, and cluster 7 was located in true Mediterranean climates with winter production. This result agrees with the patterns reported by Khazaei et al. (2016) and their distribution in the Americas since cluster 8 is cultivated in North America and cluster 7 in South America regions with a Mediterranean climate.

Clusters 7A and 8A showed similar values of private alleles to the accessions of the same clusters from the Mediterranean. Nonetheless, a significant decrease in allelic richness occurred in cluster 7A (South America; Figure S3a). The introductions of lentils in these two areas of the Americas have different characteristics. Lentil was introduced by the Spanish to South America in the XVI century via Chile (Khazaei et al., 2016). In Spain, 7 is the predominant cluster, the same as in Chile and Argentina. It is likely that a strong genetic bottleneck resulted from this introduction, leading to a decline in variation. In the case of North America (cluster 8A), a strong reduction in allelic richness was not observed (Figure S3a). The introduction of lentil in this region is more recent since it was first introduced into the United States in the 1930s, and then into the temperate prairies of this region in the 1960s (Muehlbauer et al., 1995). Contrary to our results, Khazaei et al. (2016) reported low genetic diversity in Canadian lines. In Canada, most of the registered varieties are related to the first two cultivars used to start the lentil production: Laird (Slinkard & Bhatty, 1979) and Eston (Slinkard, 1981). However, the Canadian breeding program has integrated variation from different provenances, leading to the recovery of genetic diversity and a reduction in private variation. We believe this is consistent with the activities of lentil breeders, particularly Fred Muelbauer at Washington State and Albert Vandenberg in Saskatchewan (with similar efforts from William Erskine at ICARDA for other regions) who were very intentional about making wide crosses to increase diversity in lentil.

The genomic distribution of Tajima's D can be used to infer demographic processes (Tajima, 1989; Fu, 1997). A particularly high peak in the negative part of the Tajima's D distribution was observed for clusters 3 (Iran) and 4 (South Asia), which might suggest a demographic expansion despite the low genetic diversity in these clusters. Possibly not enough time has occurred to accumulate new variation through mutation that counteracts the diversity lost during the spread bottleneck. On the other hand, clusters 2 (Middle East) and 5 (Eastern Africa) showed a peak on the positive side of the Tajima's D distribution, indicating a demographic bottleneck, despite the high genetic variation found in those clusters. A demographic reduction (suggested by Tajima's D distribution) accompanied by high diversity can at least partially be explained if admixture between clusters of cultivated lentils has taken place. Furthermore, accessions from Eastern Africa and nearby areas (cluster 5) had high levels of differentiation in terms of both *F*_ST_ (Figure S4) and *V*_ST_ (Figure S7b), and its high level of private variation stands out (Figure S3b). A scenario that can lead to high genetic diversity, high differentiation, and a possible genetic bottleneck is the introduction of alleles from a wild relative through gene flow. Hybridization between *L. culinaris* and its closest wild relative *L. orientalis* can easily occur, but the crossability with other relatives is highly dependent on the involved parents and is less common (Guerra‐García et al., 2021). Further analyses evaluating introgression from wild relatives need to be conducted in order to estimate the possible effect that gene flow has had in the history of the lentil genetic pools, in particular in lentils from Eastern Africa, southern Arabia, Egypt, and the Southern Levant.

The population structure of the LDP and how genetic diversity is distributed, therefore, are the result of the initial variation from which the domestication started, the spread from its center of origin, the human management and cultural preferences, and the adaptation to different environmental conditions. Moreover, the results presented here agree with the key role of environmental factors, such as photoperiod and temperature, previously suggested by Summerfield et al. (1985), Khazaei et al. (2016), and Wright et al. (2021) since the clustering patterns reflect the different environmental conditions where lentil is grown.

### 4.2 SNPs and CNVs provide different information about the lentil genome

Contrasting the widespread use of SNPs in evolutionary crop studies, population genomics analyses based on CNVs are still limited. We find that SNPs and CNVs have broadly similar patterns of genetic variation in lentils, showing that CNVs are not just important because of their phenotypic effects, but they also provide information about how the genetic diversity of the populations is structured. CNVs showed patterns and information not detected with the SNPs; however, the latter showed greater resolution for population structure inferences.

Gene duplication is a mechanism by which new functions and phenotypic novelties might arise (Ohno, 1970). Despite this, most duplicated genes are eventually lost (Lynch & Conery, 2000). Several models have been proposed to explain the retention of duplicated genes, such as neofunctionalization (Ohno, 1970) and subfunctionalization (Force et al., 1999). Absolute dosage and dosage‐balance constraints, which do not involve a change in function, have also been proposed (Birchler & Veitia, 2012; Gout et al., 2010; Qian et al., 2010). These models are not mutually exclusive and can be occurring simultaneously throughout a genome. Furthermore, numerous studies have found that CNVs could be an important source of adaptive variation in crops and/or affect domestication traits (e.g. Alonge et al., 2020; Bai et al., 2016; Todesco et al., 2020), and disease resistance genes are a classic example of this (e.g. Chavan et al., 2015; Cook et al., 2012). We found that 11% of all lentil genes were affected by CNVs, but an overrepresentation of resistance genes was detected (21% of resistance genes). As many resistance genes have arisen from tandem duplications, the higher incidence is not surprising.

In most of the clusters, the “Enzyme” category was present in a higher proportion of CNV compared with its fraction in the total lentil genes. This is a wide and complex category, and it is difficult to infer the effects of the presence of CNVs in these enzymes. Two other gene categories affected by CNVs in most of the clusters were “External Stimuli Response” and “Cell Wall Organization.” All the genes found in the first category were associated with pathogen response, and the latter category was primarily related to xylan synthesis (Figure S9), which is important for cell wall integrity and increases cell wall recalcitrance to enzymatic digestion thus, it helps plants to defend against herbivores and pathogens (Faik, 2013). The overrepresentation of these last two gene categories may suggest that CNVs have played a role in adaptation and acquired resistance in different environments. In the particular case of the cluster from South Asia, which presented the highest proportion of genes affected by CNV related to external stimuli response, the transition to the subtropical climates of that region would have led to exposure to a range of new pathogens. South Asia is a center of domestication of several tropical legumes like *Vigna radiata* and *Cajanus cajan* that thrive in monsoonal conditions. Year‐round legume production may have further increased exposure to potential pathogens in South Asian lentils. By contrast, in temperate growing regions lentils are in the field for a shorter amount of time and often in regions that have a cold winter, both of which help control pathogens. Our results show that CNV approaches are needed to examine how this kind of variation is evolving.

Although CNVs are a source of variation, they affect longer regions of the genome compared with SNPs, and so, it is expected that they generally would be deleterious and subject to purifying selection, resulting in an excess of CNVs at low frequency (Katju & Bergthorsson, 2013). Our results are consistent with this prediction and a high proportion of low‐frequency CNVs was observed in the SFS compared with the expected and SNP‐based SFS. This pattern has also been reported in crops such as grapevine (Zhou et al., 2019), rice (Bai et al., 2016; Fuentes et al., 2019), and tomato (Alonge et al., 2020). The observed SNP‐based SFS showed a very different shape, since in all clusters, there was a lack of low‐frequency variants (Figure 2c). This could be reflective of the autogamous reproduction system of lentils combined with the bottlenecks associated with domestication and spread of the crop.

It is worth noting that we have no doubt underestimated CNV variation since they are less likely to occur in coding regions due to their deleterious nature (Emerson et al., 2008; Katju & Bergthorsson, 2013; Zhang et al., 2009). Despite this, we were able to describe patterns related to the genes affected by the CNVs.

### 4.3 Loci under selection in lentil

Thirty‐three genes met the condition of being detected by at least two of the selection tests. Candidate genes under selection were classified into 14 categories, none of which were among the most abundant categories in the lentil genome, and three of the candidate genes under selection were not annotated. This might suggest that the candidate genes under selection were not randomly identified, but they were reflecting regions in the genome under selection. The category with the highest proportion of candidate genes under selection was “Protein Modification” (Figure S11), comprising different types of kinases (Table S2). Among the annotated genes are a long‐chain fatty acid hydroxylase (Lcu.2RBY.7G006750), which is involved in the sporopollenin synthesis in pollen (Dobritsa et al., 2009); a component of chlorophyll B reductase (Lcu.2RBY.7G021070) that has an effect regulating the amount of chlorophyll present in the cells and partially determines the antenna size (Jia et al., 2015); the protein TIC 22 (Lcu.2RBY.3G061880), which is transported to the inner envelope membrane of chloroplasts (Herrmann, 2018); and the component FtsH12 of the translocation ATPase motor complex in the chloroplast (Lcu.2RBY.7G021140) that is associated with TIC (Kikuchi et al., 2018; Mielke et al., 2020). The last two candidate genes under selection are located in different chromosomes, therefore they are not physically linked, but both are related to the import system of the inner membrane of the chloroplast.

Regional linkage disequilibrium (LD) can be used to find signatures of selection, high LD across the genome might obscure the identification of regions under selection. Because lentil is a self‐pollinated species, it harbors long blocks of LD. Furthermore, long structural variants (SV) have been discovered in the lentil genome (Ramsay et al., 2021). These factors and the complexity and size of the lentil genome hamper the identification of the causal genes associated with important traits. Due to the long blocks of LD, it is possible that we detected genes linked to the genes under selection and not the causal genes themselves. The selection tests detected regions that overlapped, for example in chromosomes 3, 5, and 7, where the three methods detect loci under selection around the same area (Figure S11) but did not concur in a particular gene. The regions linked to the candidate genes under selection found in this study can be further examined in order to discern the causal genes.

## CONFLICT OF INTEREST

The authors declare no conflict of interest.

## Supporting information


Appendix S1

## Data Availability

Exome capture SNP data for this study are available at https://knowpulse.usask.ca/AGILE/2 under associated data sets. The scripts are available at https://github.com/AzaleaGuerra/EvoHistLentil‐CNVs‐SNPs. The README file contains further details about the script found in the repository.

## References

[eva13467-bib-0001] Alonge, M. , Wang, X. , Benoit, M. , Soyk, S. , Pereira, L. , Zhang, L. , Suresh, H. , Ramakrishnan, S. , Maumus, F. , Ciren, D. , Levy, Y. , Harel, T. H. , Shalev‐Schlosser, G. , Amsellem, Z. , Razifard, H. , Caicedo, A. L. , Tieman, D. M. , Klee, H. , Kirsche, M. , … Lippman, Z. B. (2020). Major impacts of widespread structural variation on gene expression and crop improvement in tomato. Cell, 182(1), 145–161.e23. 10.1016/j.cell.2020.05.021 32553272 PMC7354227

[eva13467-bib-0002] Arumuganathan, K. , & Earle, E. D. (1991). Nuclear DNA content of some important plant species. Plant Molecular Biology Reporter, 9, 415. 10.1007/BF02672016

[eva13467-bib-0003] Bai, Z. , Chen, J. , Liao, Y. , Wang, M. , Liu, R. , Ge, S. , Wing, R. A. , & Chen, M. (2016). The impact and origin of copy number variations in the Oryza species. BMC Genomics, 17, 261. 10.1186/s12864-016-2589-2 27025496 PMC4812662

[eva13467-bib-0004] Birchler, J. A. , & Veitia, R. A. (2012). Gene balance hypothesis: Connecting issues of dosage sensitivity across biological disciplines. Proceedings of the National Academy of Sciences of the United States of America, 109(37), 14746–14753. 10.1073/pnas.1207726109 22908297 PMC3443177

[eva13467-bib-0005] Bolger, A. M. , Lohse, M. , & Usadel, B. (2014). Trimmomatic: A flexible trimmer for Illumina sequence data. Bioinformatics, 30(15), 2114–2120. 10.1093/bioinformatics/btu170 24695404 PMC4103590

[eva13467-bib-0006] Camacho‐Villa, T. C. , Maxted, N. , Scholten, M. , & Ford‐Lloyd, B. (2005). Defining and identifying crop landraces. Plant Genetic Resources, 3, 373–384. 10.1079/pgr200591

[eva13467-bib-0007] Chavan, S. , Gray, J. , & Smith, S. M. (2015). Diversity and evolution of Rp1 rust resistance genes in four maize lines. Theoretical and Applied Genetics, 128(5), 985–998. 10.1007/s00122-015-2484-2 25805314

[eva13467-bib-0008] Cook, D. E. , Lee, T. G. , Guo, X. , Melito, S. , Wang, K. , Bayless, A. M. , Wang, J. , Hughes, T. J. , Willis, D. K. , Clemente, T. E. , Diers, B. W. , Jiang, J. , Hudson, M. E. , & Bent, A. F. (2012). Copy number variation of multiple genes at Rhg1 mediates nematode resistance in soybean. Science, 338(6111), 1206–1209. 10.1126/science.1228746 23065905

[eva13467-bib-0102] Cubero, J. I. , Perez De La Vega, M. , & Fratini, R. (2009). Origin, phylogeny, domestication and spread. In The lentil: Botany, production and uses (pp. 13–33). CABI.

[eva13467-bib-0009] Danecek, P. , Auton, A. , Abecasis, G. , Albers, C. A. , Banks, E. , DePristo, M. A. , Handsaker, R. E. , Lunter, G. , Marth, G. T. , Sherry, S. T. , McVean, G. , Durbin, R. , & 1000 Genomes Project Analysis Group . (2011). The variant call format and VCFtools. Bioinformatics, 27(15), 2156–2158. 10.1093/bioinformatics/btr330 21653522 PMC3137218

[eva13467-bib-0010] Dissanayake, R. , Braich, S. , Cogan, N. O. I. , Smith, K. , & Kaur, S. (2020). Characterization of genetic and allelic diversity amongst cultivated and wild lentil accessions for germplasm enhancement. Frontiers in Genetics, 11, 546. 10.3389/fgene.2020.00546 32587602 PMC7298104

[eva13467-bib-0011] Dobritsa, A. A. , Shrestha, J. , Morant, M. , Pinot, F. , Matsuno, M. , Swanson, R. , Møller, B. L. , & Preuss, D. (2009). CYP704B1 is a long‐chain fatty acid ω‐hydroxylase essential for sporopollenin synthesis in pollen of Arabidopsis. Plant Physiology, 151(2), 574–589. 10.1104/pp.109.144469 19700560 PMC2754625

[eva13467-bib-0012] Dorant, Y. , Cayuela, H. , Wellband, K. , Laporte, M. , Rougemont, Q. , Mérot, C. , Normandeau, E. , Rochette, R. , & Bernatchez, L. (2020). Copy number variants outperform SNPs to reveal genotype‐temperature association in a marine species. Molecular Ecology, 29(24), 4765–4782. 10.1111/mec.15565 32803780

[eva13467-bib-0013] Emerson, J. J. , Cardoso‐Moreira, M. , Borevitz, J. O. , & Long, M. (2008). Natural selection shapes genome‐wide patterns of copy‐number polymorphism in Drosophila melanogaster. Science, 320(5883), 1629–1631. 10.1126/science.1158078 18535209

[eva13467-bib-0014] Erskine, W. , Chandra, S. , Chaudhry, M. , Malik, I. A. , Sarker, A. , Sharma, B. , Tufail, M. , & Tyagi, M. C. (1998). A bottleneck in lentil: Widening its genetic base in South Asia. Euphytica, 101(2), 207–211. 10.1023/A:1018306723777

[eva13467-bib-0015] Evanno, G. , Regnaut, S. , & Goudet, J. (2005). Detecting the number of clusters of individuals using the software STRUCTURE: A simulation study. Molecular Ecology, 14(8), 2611–2620. 10.1111/j.1365-294X.2005.02553.x 15969739

[eva13467-bib-0016] Faik, A. (2013). “Plant Cell Wall structure‐pretreatment” the critical relationship in biomass conversion to fermentable sugars. In T. Gu (Ed.), Green biomass pretreatment for biofuels production (pp. 1–30). Springer. 10.1007/978-94-007-6052-3_1

[eva13467-bib-0017] FAOSTAT . 2020. http://www.fao.org/faostat/en/#home

[eva13467-bib-0018] Ferguson, M. E. , Ford‐Lloyd, B. V. , Robertson, L. D. , Maxted, N. , & Newbury, H. J. (1998). Mapping the geographical distribution of genetic variation in the genusLensfor the enhanced conservation of plant genetic diversity. Molecular Ecology, 7, 1743–1755. 10.1046/j.1365-294x.1998.00513.x

[eva13467-bib-0019] Foll, M. , & Gaggiotti, O. (2008). A genome‐scan method to identify selected loci appropriate for both dominant and codominant markers: A Bayesian perspective. Genetics, 180(2), 977–993. 10.1534/genetics.108.092221 18780740 PMC2567396

[eva13467-bib-0020] Force, A. , Lynch, M. , Pickett, F. B. , Amores, A. , Yan, Y. L. , & Postlethwait, J. (1999). Preservation of duplicate genes by complementary, degenerative mutations. Genetics, 151(4), 1531–1545. 10101175 10.1093/genetics/151.4.1531 PMC1460548

[eva13467-bib-0021] Freeman, J. L. , Perry, G. H. , Feuk, L. , Redon, R. , McCarroll, S. A. , Altshuler, D. M. , Aburatani, H. , Jones, K. W. , Tyler‐Smith, C. , Hurles, M. E. , Carter, N. P. , Scherer, S. W. , & Lee, C. (2006). Copy number variation: New insights in genome diversity. Genome Research, 16(8), 949–961. 10.1101/gr.3677206 16809666

[eva13467-bib-0022] Fu, Y. X. (1997). Statistical tests of neutrality of mutations against population growth, hitchhiking and background selection. Genetics, 147(2), 915–925. 9335623 10.1093/genetics/147.2.915 PMC1208208

[eva13467-bib-0023] Fuentes, R. R. , Chebotarov, D. , Duitama, J. , Smith, S. , De la Hoz, J. F. , Mohiyuddin, M. , Wing, R. A. , McNally, K. L. , Tatarinova, T. , Grigoriev, A. , Mauleon, R. , & Alexandrov, N. (2019). Structural variants in 3000 rice genomes. Genome Research, 29(5), 870–880. 10.1101/gr.241240.118 30992303 PMC6499320

[eva13467-bib-0024] Gaut, B. S. , Seymour, D. K. , Liu, Q. , & Zhou, Y. (2018). Demography and its effects on genomic variation in crop domestication. Nature Plants, 4(8), 512–520. 10.1038/s41477-018-0210-1 30061748

[eva13467-bib-0025] Gout, J. F. , Kahn, D. , Duret, L. , & Paramecium Post‐Genomics Consortium . (2010). The relationship among gene expression, the evolution of gene dosage, and the rate of protein evolution. PLoS Genetics, 6, e1000944. 10.1371/journal.pgen.1000944 20485561 PMC2869310

[eva13467-bib-0026] Gross, B. L. (2012). Genetic and phenotypic divergence of homoploid hybrid species from parental species. Heredity, 108(3), 157–158. 10.1038/hdy.2011.80 21915149 PMC3282403

[eva13467-bib-0027] Guerra‐García, A. , Gioia, T. , von Wettberg, E. , Logozzo, G. , Papa, R. , Bitocchi, E. , & Bett, K. E. (2021). Intelligent characterization of lentil genetic resources: Evolutionary history, genetic diversity of germplasm, and the need for well‐represented collections. Current Protocols, 1(5), e134. 10.1002/cpz1.134 34004055

[eva13467-bib-0028] Haile, T. A. , Heidecker, T. , Wright, D. , Neupane, S. , Ramsay, L. , Vandenberg, A. , & Bett, K. E. (2020). Genomic selection for lentil breeding: Empirical evidence. The Plant Genome, 13(1), e20002. 10.1002/tpg2.20002 33016638 PMC12807041

[eva13467-bib-0029] Hardigan, M. A. , Crisovan, E. , Hamilton, J. P. , Kim, J. , Laimbeer, P. , Leisner, C. P. , Manrique‐Carpintero, N. C. , Newton, L. , Pham, G. M. , Vaillancourt, B. , Yang, X. , Zeng, Z. , Douches, D. S. , Jiang, J. , Veilleux, R. E. , & Buell, C. R. (2016). Genome reduction uncovers a large dispensable genome and adaptive role for copy number variation in asexually propagated Solanum tuberosum. The Plant Cell, 28(2), 388–405. 10.1105/tpc.15.00538 26772996 PMC4790865

[eva13467-bib-0030] Herrmann, J. M. (2018). A force‐generating machine in the Plant's powerhouse: A pulling AAA‐ATPase motor drives protein translocation into chloroplasts. Plant Cell, 30(11), 2646–2647. 10.1105/tpc.18.00751 30309903 PMC6305984

[eva13467-bib-0031] Hoban, S. , Kelley, J. L. , Lotterhos, K. E. , Antolin, M. F. , Bradburd, G. , Lowry, D. B. , Poss, M. L. , Reed, L. K. , Storfer, A. , & Whitlock, M. C. (2016). Finding the genomic basis of local adaptation: Pitfalls, practical solutions, and future directions. The American Naturalist, 188(4), 379–397. 10.1086/688018 PMC5457800 27622873

[eva13467-bib-0032] Idrissi, O. , Udupa, S. M. , Houasli, C. , De Keyser, E. , Van Damme, P. , & De Riek, J. (2015). Genetic diversity analysis of Moroccan lentil (*Lens culinaris* Medik.) landraces using simple sequence repeat and amplified fragment length polymorphisms reveals functional adaptation towards agro‐environmental origins. Plant Breeding, 134, 322–332. 10.1111/pbr.12261

[eva13467-bib-0033] Jia, T. , Ito, H. , & Tanaka, A. (2015). The chlorophyll b reductase NOL participates in regulating the antenna size of photosystem II in Arabidopsis Thaliana. Procedia Chemistry, 14, 422–427. 10.1016/j.proche.2015.03.057

[eva13467-bib-0034] Katju, V. , & Bergthorsson, U. (2013). Copy‐number changes in evolution: Rates, fitness effects and adaptive significance. Frontiers in Genetics, 4, 273. 10.3389/fgene.2013.00273 24368910 PMC3857721

[eva13467-bib-0035] Khazaei, H. , Caron, C. T. , Fedoruk, M. , Diapari, M. , Vandenberg, A. , Coyne, C. J. , McGee, R. , & Bett, K. E. (2016). Genetic diversity of cultivated lentil (*Lens culinaris* Medik.) and its relation to the World's Agro‐ecological zones. Frontiers in Plant Science, 7, 1093. 10.3389/fpls.2016.01093 27507980 PMC4960256

[eva13467-bib-0036] Kikuchi, S. , Asakura, Y. , Imai, M. , Nakahira, Y. , Kotani, Y. , Hashiguchi, Y. , Nakai, Y. , Takafuji, K. , Bédard, J. , Hirabayashi‐Ishioka, Y. , Mori, H. , Shiina, T. , & Nakai, M. (2018). A Ycf2‐FtsHi heteromeric AAA‐ATPase complex is required for chloroplast protein import. The Plant Cell, 30(11), 2677–2703. 10.1105/tpc.18.00357 30309901 PMC6305978

[eva13467-bib-0037] Knaus, B. J. , Tabima, J. F. , Shakya, S. K. , Judelson, H. S. , & Grünwald, N. J. (2020). Genome‐wide increased copy number is associated with emergence of dominant clones of the Irish potato famine pathogen *Phytophthora infestans* . MBio, 11(3), e00326‐20. 10.1128/mBio.00326-20 32576669 PMC7315116

[eva13467-bib-0038] Ladizinsky, G. (1979). The origin of lentil and its wild genepool. Euphytica, 28(1), 179–187. 10.1007/BF00029189

[eva13467-bib-0039] Langmead, B. , & Salzberg, S. L. (2012). Fast gapped‐read alignment with bowtie 2. Nature Methods, 9(4), 357–359. 10.1038/nmeth.1923 22388286 PMC3322381

[eva13467-bib-0040] Li, H. , Handsaker, B. , Wysoker, A. , Fennell, T. , Ruan, J. , Homer, N. , Marth, G. , Abecasis, G. , Durbin, R. , & 1000 Genome Project Data Processing Subgroup . (2009). The sequence alignment/map format and SAMtools. Bioinformatics, 25(16), 2078–2079. 10.1093/bioinformatics/btp352 19505943 PMC2723002

[eva13467-bib-0041] Liber, M. , Duarte, I. , Maia, A. T. , & Oliveira, H. R. (2021). The history of lentil (*Lens culinaris* subsp. *culinaris*) domestication and spread as revealed by genotyping‐by‐sequencing of wild and landrace accessions. Frontiers in Plant Science, 12, 628439. 10.3389/fpls.2021.628439 33841458 PMC8030269

[eva13467-bib-0042] Lombardi, M. , Materne, M. , Cogan, N. O. I. , Rodda, M. , Daetwyler, H. D. , Slater, A. T. , Forster, J. W. , & Kaur, S. (2014). Assessment of genetic variation within a global collection of lentil (*Lens culinaris* Medik.) cultivars and landraces using SNP markers. BMC Genetics, 15(1), 150. 10.1186/s12863-014-0150-3 25540077 PMC4300608

[eva13467-bib-0043] Luu, K. , Bazin, E. , & Blum, M. G. B. (2017). Pcadapt: An R package to perform genome scans for selection based on principal component analLuis Eguiarteysis. Molecular Ecology Resources, 17(1), 67–77. 10.1111/1755-0998.12592 27601374

[eva13467-bib-0044] Lye, Z. N. , & Purugganan, M. D. (2019). Copy number variation in domestication. Trends in Plant Science, 24(4), 352–365. 10.1016/j.tplants.2019.01.003 30745056

[eva13467-bib-0045] Lynch, M. , & Conery, J. S. (2000). The evolutionary fate and consequences of duplicate genes. Science, 290(5494), 1151–1155. 11073452 10.1126/science.290.5494.1151

[eva13467-bib-0046] McKenna, A. , Hanna, M. , Banks, E. , Sivachenko, A. , Cibulskis, K. , Kernytsky, A. , Garimella, K. , Altshuler, D. , Gabriel, S. , Daly, M. , & DePristo, M. A. (2010). The genome analysis toolkit: A MapReduce framework for analyzing next‐generation DNA sequencing data. Genome Research, 20(9), 1297–1303. 10.1101/gr.107524.110 20644199 PMC2928508

[eva13467-bib-0047] Mercenaro, L. , Nieddu, G. , Porceddu, A. , Pezzotti, M. , & Camiolo, S. (2017). Sequence polymorphisms and structural variations among four grapevine (*Vitis vinifera* L.) cultivars representing Sardinian agriculture. Frontiers in Plant Science, 8, 1279. 10.3389/fpls.2017.01279 28775732 PMC5517397

[eva13467-bib-0048] Mercer, K. L. , & Perales, H. R. (2010). Evolutionary response of landraces to climate change in centers of crop diversity. Evolutionary Applications, 3, 480–493. 10.1111/j.1752-4571.2010.00137.x 25567941 PMC3352508

[eva13467-bib-0049] Mielke, K. , Wagner, R. , Mishra, L. S. , Demir, F. , Perrar, A. , Huesgen, P. F. , & Funk, C. (2020). Abundance of metalloprotease FtsH12 modulates chloroplast development in *Arabidopsis thaliana* . Journal of Experimental Botany, 72(9), 3455–3473. 10.1093/jxb/eraa550 PMC8042743 33216923

[eva13467-bib-0050] Morrell, P. L. , Buckler, E. S. , & Ross‐Ibarra, J. (2012). Crop genomics: Advances and applications. Nature Reviews Genetics, 13, 85–96. 10.1038/nrg3097 22207165

[eva13467-bib-0051] Muehlbauer, F. J. , Kaiser, W. J. , Clement, S. L. , & Summerfield, R. J. (1995). Production and breeding of lentil. Advances in Agronomy, 54, 283–332.

[eva13467-bib-0052] Ogutcen, E. , Ramsay, L. , von Wettberg, E. B. , & Bett, K. E. (2018). Capturing variation in (Fabaceae): Development and utility of an exome capture array for lentil. Applications in Plant Sciences, 6(7), e01165. 10.1002/aps3.1165 30131907 PMC6055568

[eva13467-bib-0053] Ohno, S. (1970). Evolution by gene duplication. Springer. 10.1007/978-3-642-86659-3

[eva13467-bib-0054] Pavan, S. , Bardaro, N. , Fanelli, V. , Marcotrigiano, A. R. , Mangini, G. , Taranto, F. , Catalano, D. , Montemurro, C. , de Giovanni, C. , Lotti, C. , & Ricciardi, L. (2019). Genotyping by sequencing of cultivated lentil (*Lens culinaris* Medik.) highlights population structure in the Mediterranean Gene Pool associated with geographic patterns and phenotypic variables. Frontiers in Genetics, 10, 872. 10.3389/fgene.2019.00872 31620173 PMC6759463

[eva13467-bib-0055] Qian, W. , Liao, B.‐Y. , Chang, A. Y.‐F. , & Zhang, J. (2010). Maintenance of duplicate genes and their functional redundancy by reduced expression. Trends in Genetics, 26(10), 425–430. 10.1016/j.tig.2010.07.002 20708291 PMC2942974

[eva13467-bib-0056] R Core Team . (2020). R: A language and environment for statistical computing. R Foundation for Statistical Computing. https://www.R‐project.org/

[eva13467-bib-0100] Raj, A. , Stephens, M. , & Pritchard, J. K. (2014). fastSTRUCTURE: Variational inference of population structure in large SNP data sets. Genetics, 197(2), 573–589. 24700103 10.1534/genetics.114.164350 PMC4063916

[eva13467-bib-0057] Ramsay, L. , Koh, C. S. , Kagale, S. , Gao, D. , Kaur, S. , Haile, T. , Gela T. S. , Chen L. A. , Cao Z. , Konkin D. J. , Toegelová H. , Doležel J. , Rosen B. D. , Stonehouse R. , Humann J. L. , Main D. , Coyne C. J. , McGee R. J. , Cook D. R. , … Bett K. E. (2021). Genomic rearrangements have consequences for introgression breeding as revealed by genome assemblies of wild and cultivated lentil species (p. 2021.07.23.453237). 10.1101/2021.07.23.453237

[eva13467-bib-0058] Redon, R. , Ishikawa, S. , Fitch, K. R. , Feuk, L. , Perry, G. H. , Andrews, T. D. , Fiegler, H. , Shapero, M. H. , Carson, A. R. , Chen, W. , Cho, E. K. , Dallaire, S. , Freeman, J. L. , González, J. R. , Gratacòs, M. , Huang, J. , Kalaitzopoulos, D. , Komura, D. , JR, M. D. , … Hurles, M. E. (2006). Global variation in copy number in the human genome. Nature, 444(7118), 444–454. 10.1038/nature05329 17122850 PMC2669898

[eva13467-bib-0059] Rinker, D. C. , Specian, N. K. , Zhao, S. , & Gibbons, J. G. (2019). Polar bear evolution is marked by rapid changes in gene copy number in response to dietary shift. Proceedings of the National Academy of Sciences of the United States of America, 116(27), 13446–13451. 10.1073/pnas.1901093116 31209046 PMC6613075

[eva13467-bib-0060] Schwacke, R. , Ponce‐Soto, G. Y. , Krause, K. , Bolger, A. M. , Arsova, B. , Hallab, A. , Gruden, K. , Stitt, M. , Bolger, M. E. , & Usadel, B. (2019). MapMan4: A refined protein classification and annotation framework applicable to multi‐omics data analysis. Molecular Plant, 12, 879–892. 10.1016/j.molp.2019.01.003 30639314

[eva13467-bib-0061] Slinkard, A. E. (1981). Eston lentil. Canadian Journal of Plant Science, 61(3), 733–734. 10.4141/cjps81-104

[eva13467-bib-0062] Slinkard, A. E. , & Bhatty, R. S. (1979). Laird lentil. Canadian Journal of Plant Science, 59(2), 503–504. 10.4141/cjps79-079

[eva13467-bib-0063] Summerfield, R. J. , Roberts, E. H. , Erskine, W. , & Ellis, R. H. (1985). Effects of temperature and photoperiod on flowering in lentils (*Lens culinaris* medic.). Annals of Botany, 56(5), 659–671. 10.1093/oxfordjournals.aob.a087055

[eva13467-bib-0064] Sun, S. , Zhou, Y. , Chen, J. , Shi, J. , Zhao, H. , Zhao, H. , Song, W. , Zhang, M. , Cui, Y. , Dong, X. , Liu, H. , Ma, X. , Jiao, Y. , Wang, B. , Wei, X. , Stein, J. C. , Glaubitz, J. C. , Lu, F. , Yu, G. , … Lai, J. (2018). Extensive intraspecific gene order and gene structural variations between Mo17 and other maize genomes. Nature Genetics, 50(9), 1289–1295. 10.1038/s41588-018-0182-0 30061735

[eva13467-bib-0065] Swanson‐Wagner, R. A. , Eichten, S. R. , Kumari, S. , Tiffin, P. , Stein, J. C. , Ware, D. , & Springer, N. M. (2010). Pervasive gene content variation and copy number variation in maize and its undomesticated progenitor. Genome Research, 20(12), 1689–1699. 10.1101/gr.109165.110 21036921 PMC2989995

[eva13467-bib-0066] Szpiech, Z. A. , Jakobsson, M. , & Rosenberg, N. A. (2008). ADZE: A rarefaction approach for counting alleles private to combinations of populations. Bioinformatics, 24(21), 2498–2504. 10.1093/bioinformatics/btn478 18779233 PMC2732282

[eva13467-bib-0067] Tajima, F. (1989). Statistical method for testing the neutral mutation hypothesis by DNA polymorphism. Genetics, 123(3), 585–595. 2513255 10.1093/genetics/123.3.585 PMC1203831

[eva13467-bib-0068] Tarasov, A. , Vilella, A. J. , Cuppen, E. , Nijman, I. J. , & Prins, P. (2015). Sambamba: Fast processing of NGS alignment formats. Bioinformatics, 31(12), 2032–2034. 10.1093/bioinformatics/btv098 25697820 PMC4765878

[eva13467-bib-0069] Todesco, M. , Owens, G. L. , Bercovich, N. , Légaré, J.‐S. , Soudi, S. , Burge, D. O. , Huang, K. , Ostevik, K. L. , EBM, D. , Imerovski, I. , Lande, K. , Pascual‐Robles, M. A. , Nanavati, M. , Jahani, M. , Cheung, W. , Staton, S. E. , Muños, S. , Nielsen, R. , Donovan, L. A. , … Rieseberg, L. H. (2020). Massive haplotypes underlie ecotypic differentiation in sunflowers. Nature, 584(7822), 602–607. 10.1038/s41586-020-2467-6 32641831

[eva13467-bib-0070] Warschefsky, E. , Penmetsa, R. V. , Cook, D. R. , & von Wettberg, E. J. B. (2014). Back to the wilds: Tapping evolutionary adaptations for resilient crops through systematic hybridization with crop wild relatives. American Journal of Botany, 101(10), 1791–1800. 10.3732/ajb.1400116 25326621

[eva13467-bib-0101] Weir, B. S. , & Cockerham, C. C. (1984). Estimating F‐statistics for the analysis of population structure. Evolution, 38(6), 1358–1370. 10.1111/j.1558-5646.1984.tb05657.x 28563791

[eva13467-bib-0071] Wright, D. M. , Neupane, S. , Heidecker, T. , Haile, T. A. , Chan, C. , Coyne, C. J. , McGee, R. J. , Udupa, S. , Henkrar, F. , Barilli, E. , Rubiales, D. , Gioia, T. , Logozzo, G. , Marzario, S. , Mehra, R. , Sarker, A. , Dhakal, R. , Anwar, B. , Sarkar, D. , … Bett, K. E. (2021). Understanding photothermal interactions will help expand production range and increase genetic diversity of lentil (*Lens culinaris* Medik.). Plants, People, Planet, 3, 171–181. 10.1002/ppp3.10158

[eva13467-bib-0072] Zhang, F. , Gu, W. , Hurles, M. E. , & Lupski, J. R. (2009). Copy number variation in human health, disease, and evolution. Annual Review of Genomics and Human Genetics, 10, 451–481. 10.1146/annurev.genom.9.081307.164217 PMC4472309 19715442

[eva13467-bib-0073] Zheng, X. , Levine, D. , Shen, J. , Gogarten, S. M. , Laurie, C. , & Weir, B. S. (2012). A high‐performance computing toolset for relatedness and principal component analysis of SNP data. Bioinformatics, 28(24), 3326–3328. 10.1093/bioinformatics/bts606 23060615 PMC3519454

[eva13467-bib-0074] Zhou, Y. , Minio, A. , Massonnet, M. , Solares, E. , Lv, Y. , Beridze, T. , Cantu, D. , & Gaut, B. S. (2019). The population genetics of structural variants in grapevine domestication. Nature Plants, 5(9), 965–979. 10.1038/s41477-019-0507-8 31506640

