# Supplementary material for: An evolutionary look into the history of lentil reveals unexpected diversity
Source: Evol Appl. 2022 Aug 21;15(8):1313–25. doi: 10.1111/eva.13467 (PMC9423085; doi:10.1111/eva.13467)
Supplement: Supplementary file 1 — Appendix S1 [file EVA-15-1313-s001.docx]

**Unexpected diversity in lentils: An evolutionary look back into the history of lentil using exome capture**

Supplementary material

**Table S1.** Details of the genotypes that integrate the Lentil Diversity Panel.

[**https://github.com/derekmichaelwright/AGILE_LDP_Phenology/blob/master/Supplemental_Table_01.csv**](https://github.com/derekmichaelwright/AGILE_LDP_Phenology/blob/master/Supplemental_Table_01.csv)


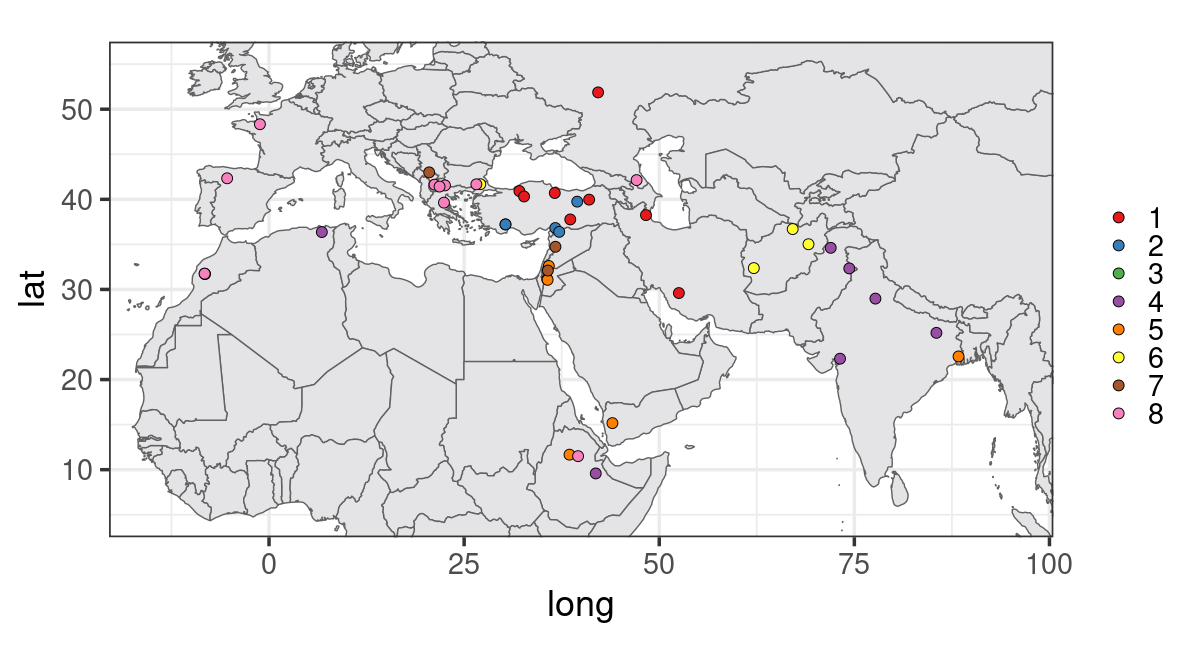


Figure S1. Geographic distribution of the used lentil accessions from Asia, Europe and Africa. Colours show the genetic groups. An important proportion of the accessions did not have geographic coordinates associated.

**Figure S2.** a) Segregating sites count within each genetic group of the Lentil Diversity Panel (LDP). The numbers above the columns indicate the number of accessions included b) CNV loci count according to the genetic clusters. The number on top of the columns shows the lentil accessions within the clusters.
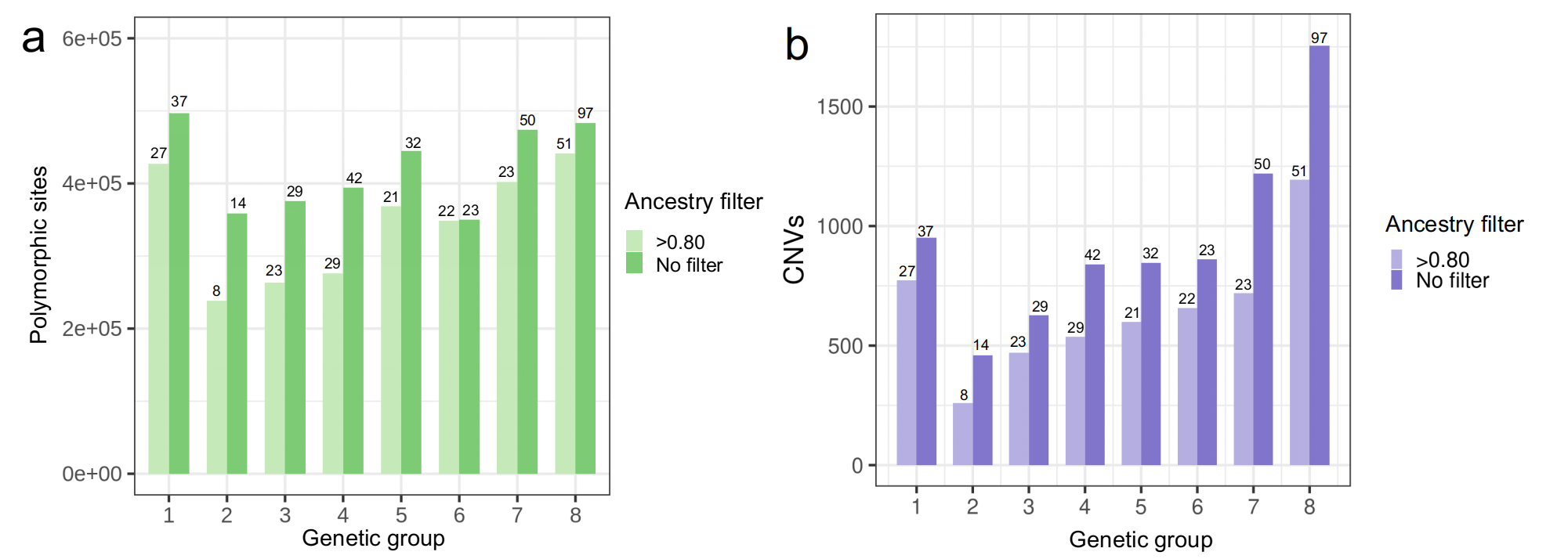


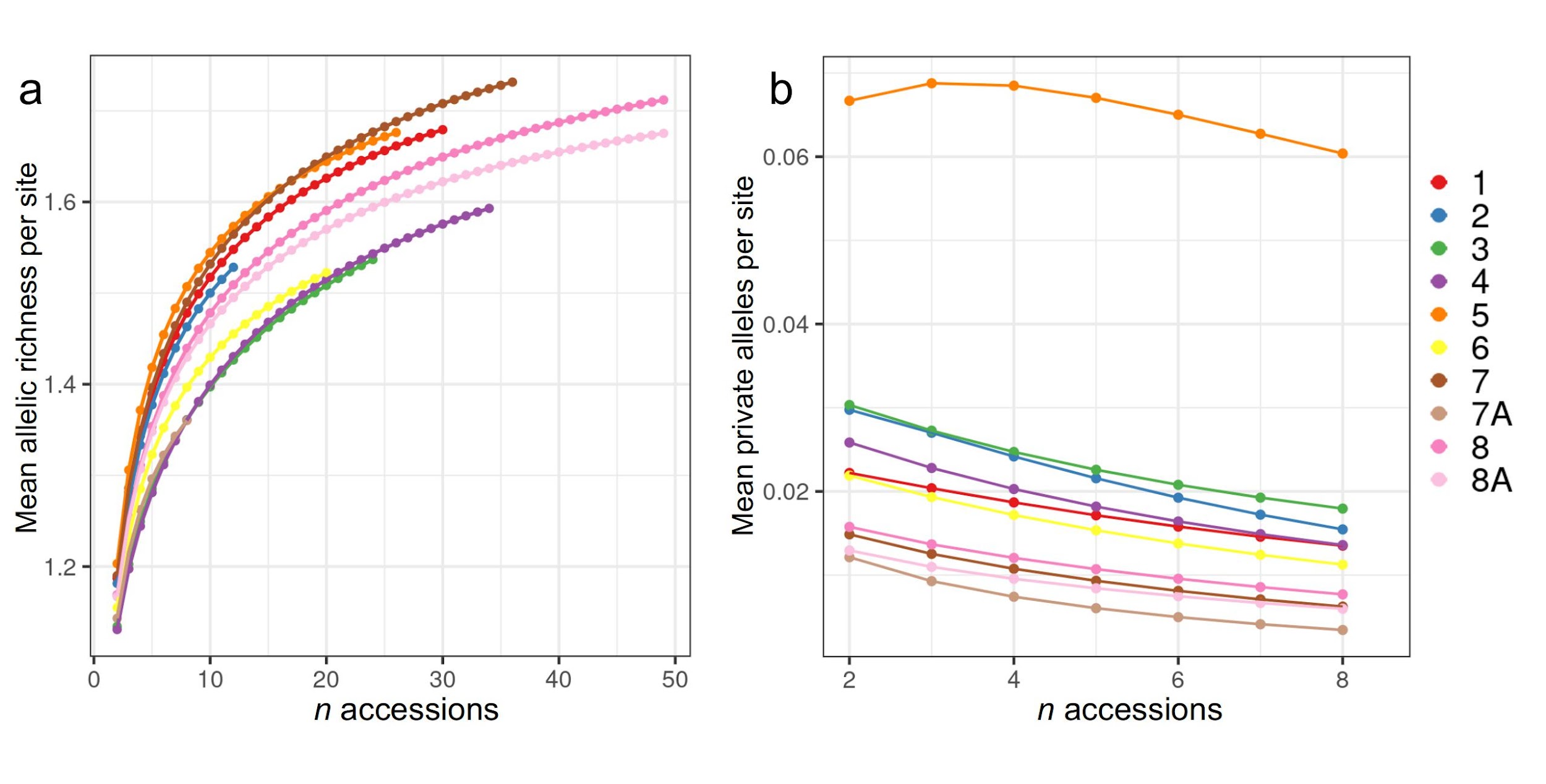


**Figure S3.** a) Mean allelic richness per site and b) mean private alleles per site estimated with AZDE for each population.


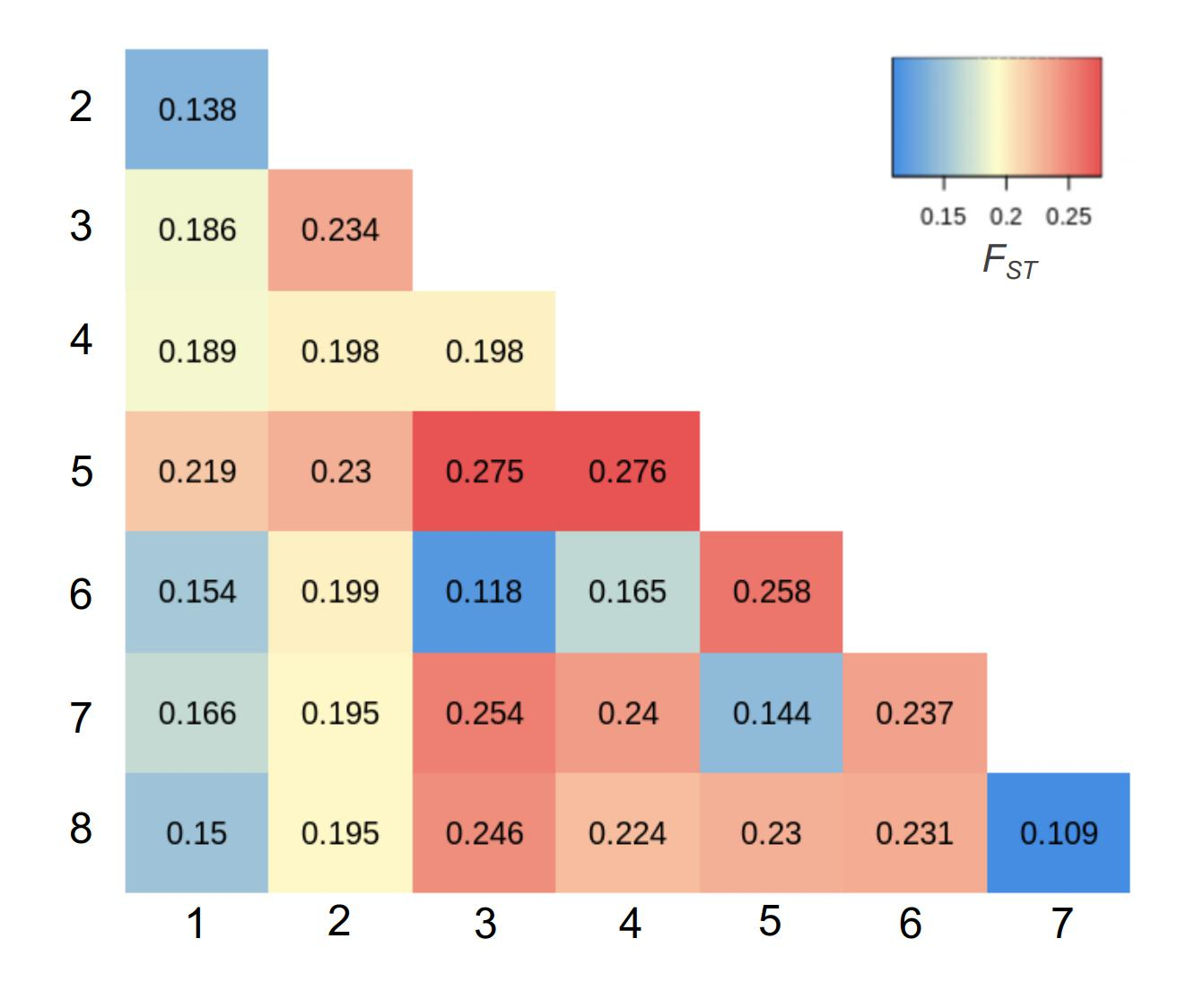


**Figure S4**. Heatmap representing the pair *F*_ST_ between the lentil clusters.


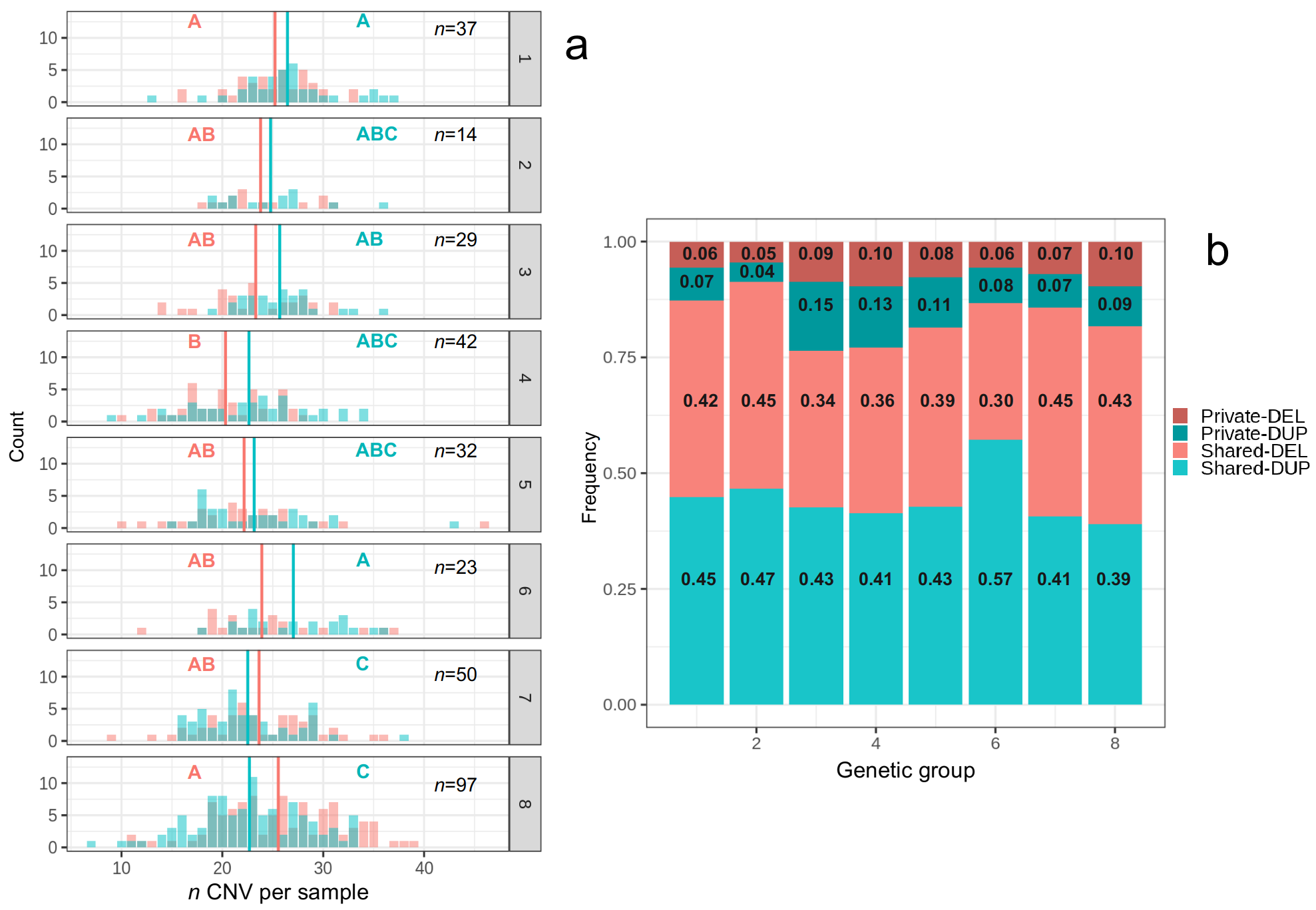


**Figure S5**. a) Count and b) proportion of private and shared CNV loci according to the lentil genetic groups. The letters in a) show groups that are statistically different and are decreasingly ordered.


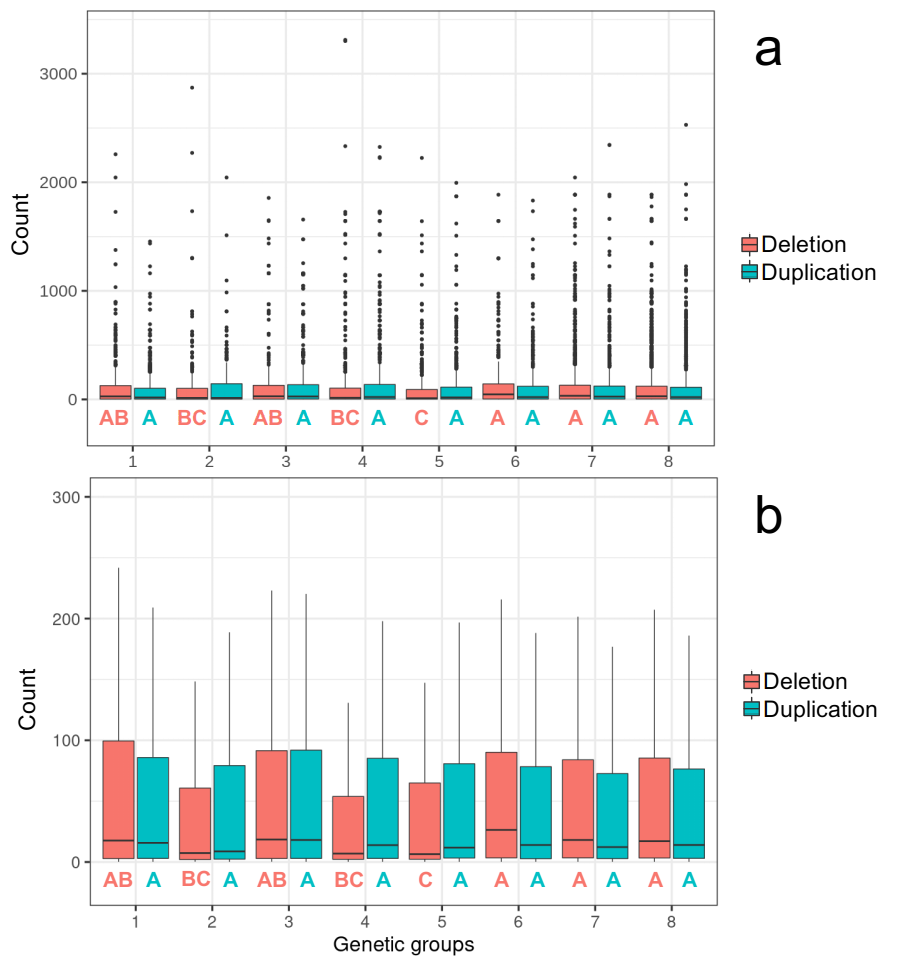


**Figure S6**. Length of the CNV found in the LDP according to the eight genetic groups. a) includes the outliers, meanwhile in b) outliers were removed. Letters show the statistical differences among the groups.


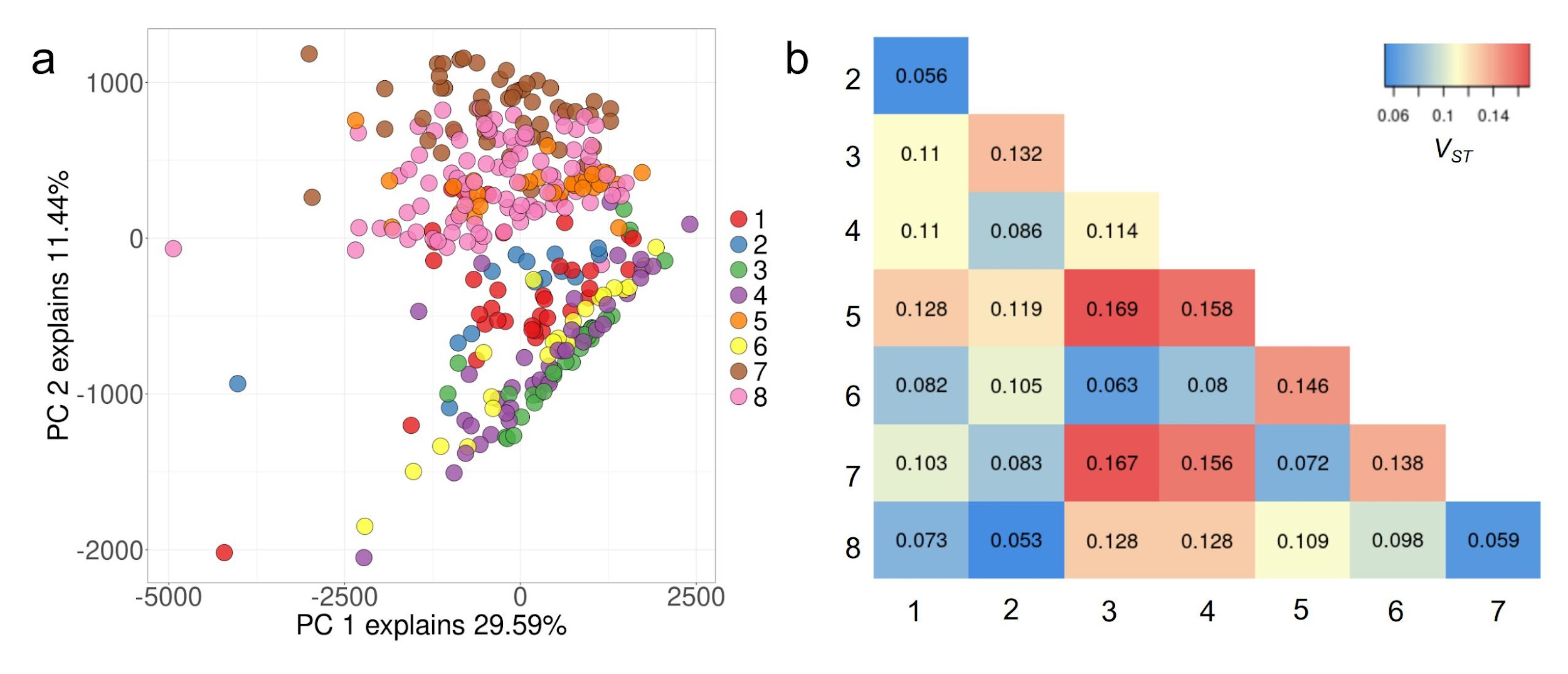


**Figure S7**. a) PCA plot showing the first two principal components inferred from the CNV regions using the normalized depth. b) Heatmap of *V_ST_*, which quantifies population differentiation through the variance in the normalized depth, estimated from CNV loci.


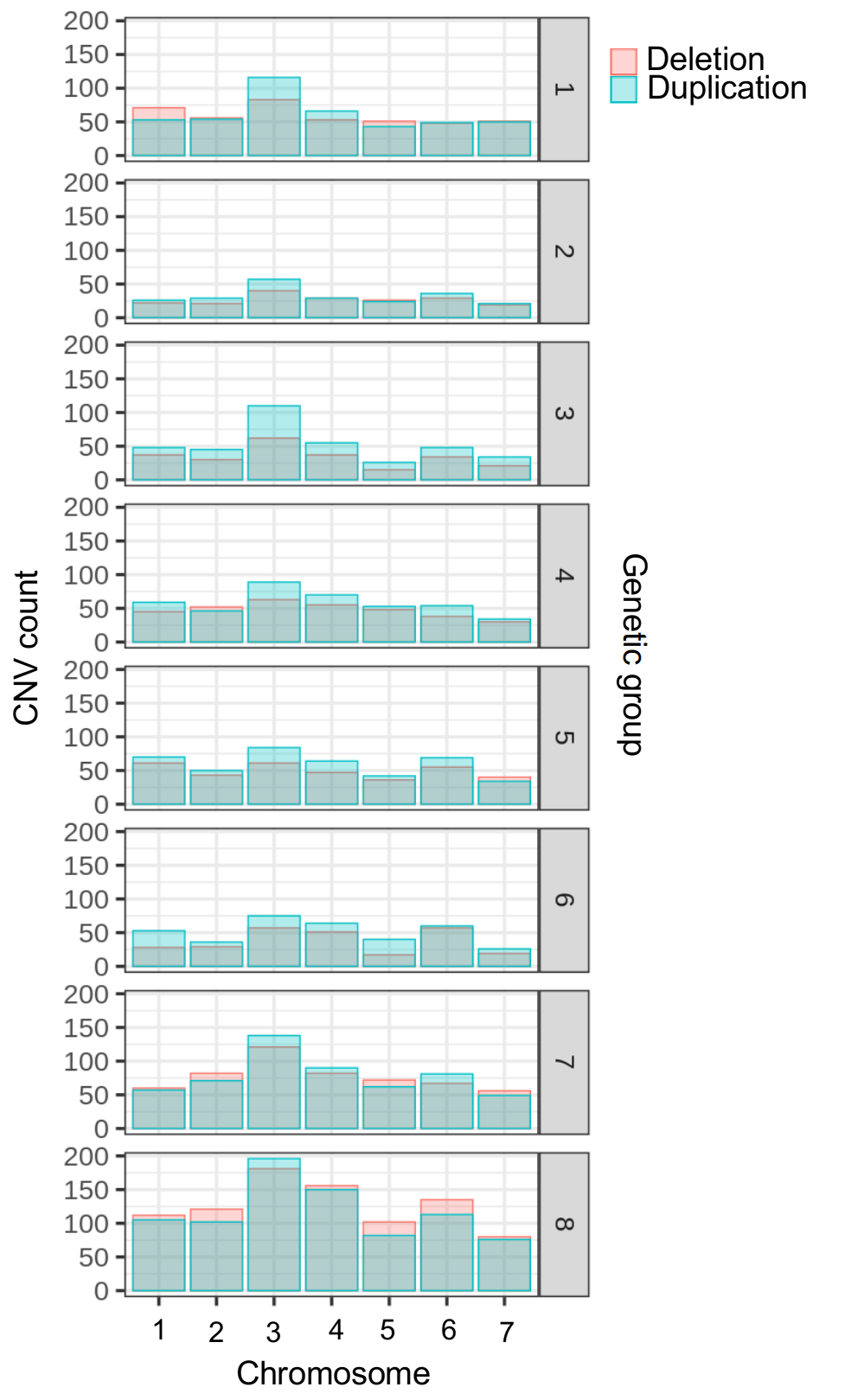


**Figure S8**. CNVs distribution across the lentil chromosomes and split according to the genetic clusters.


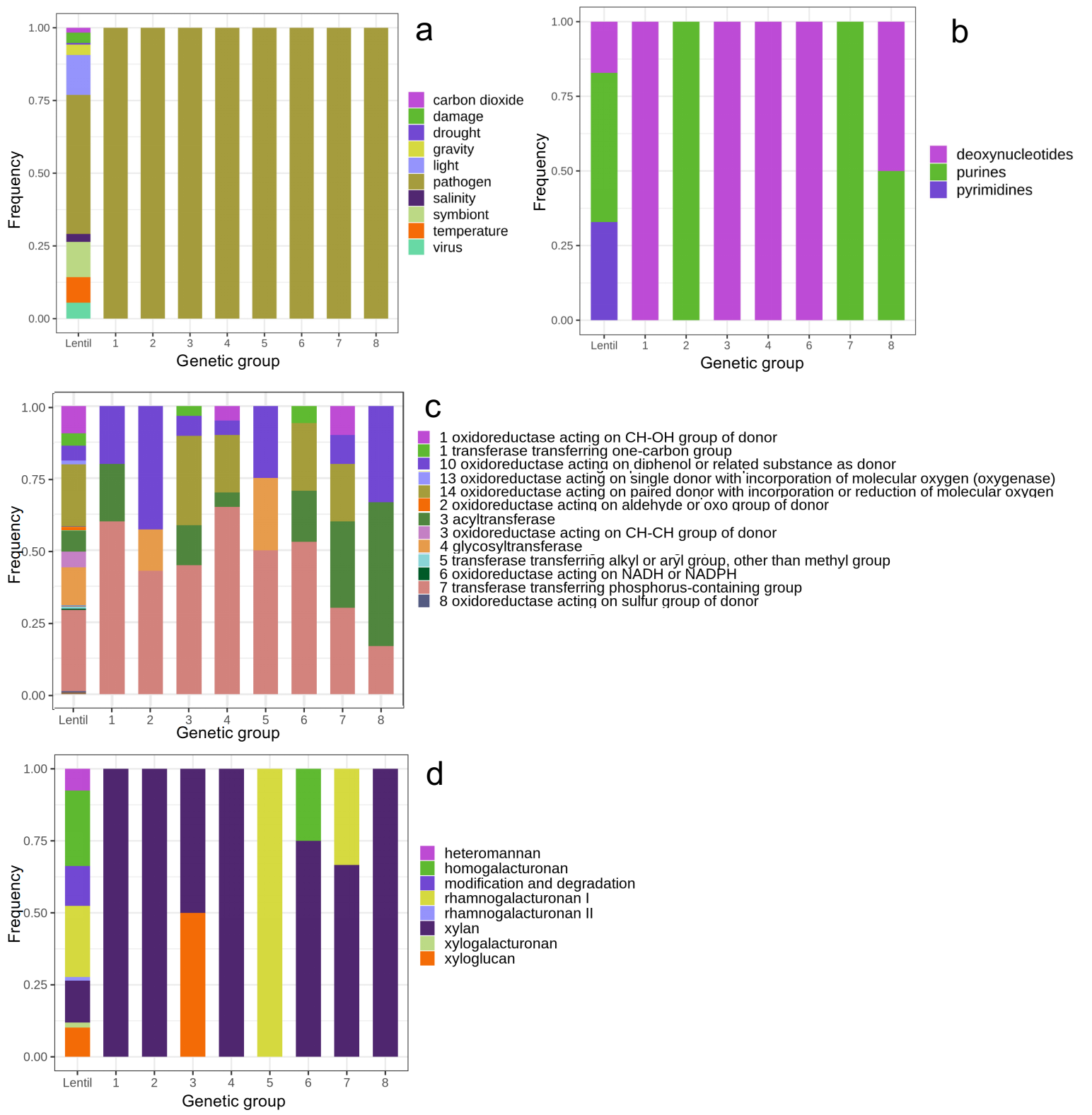


**Figure S9**. MapMan subclassification of the genes in CNVs found in a) stimuli response, b) nucleotide metabolism, c) enzyme categories, and d) cell wall organization.


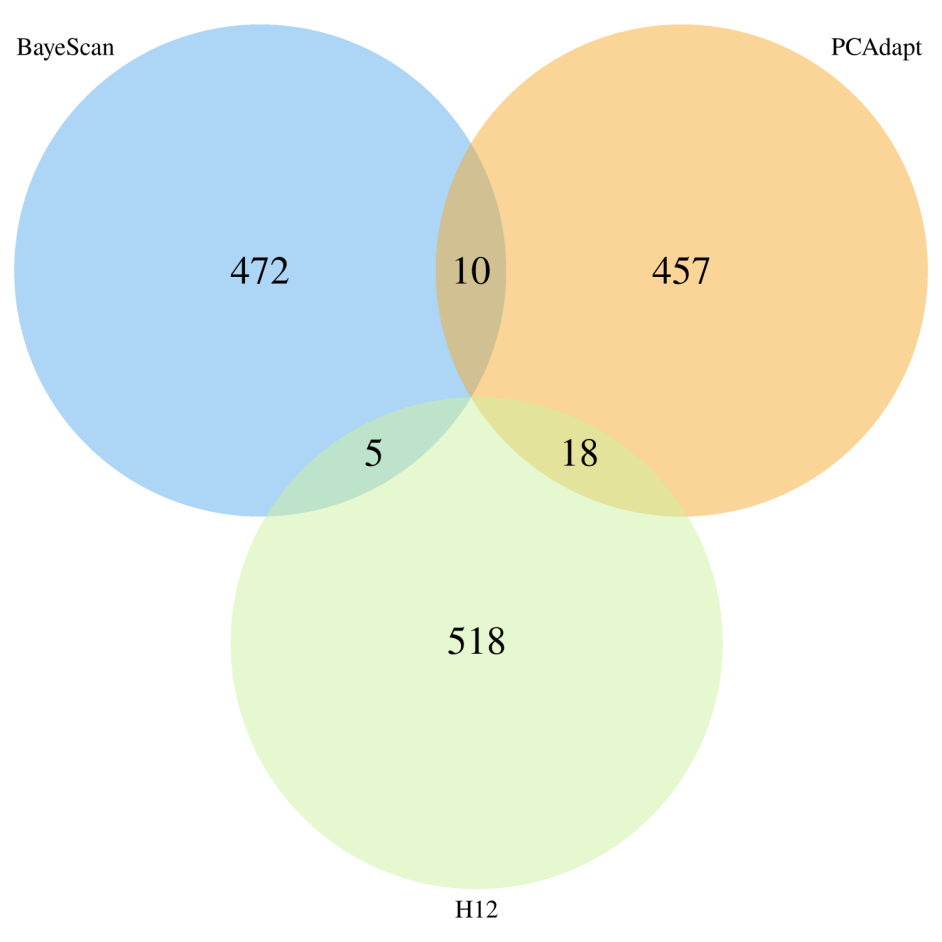


**Figure S10**. Venn diagram showing the genes detected using pcadapt (orange), Bayescan (blue) and SelectionHapStats (green). Genes detected by at least two methods were considered as true candidates.


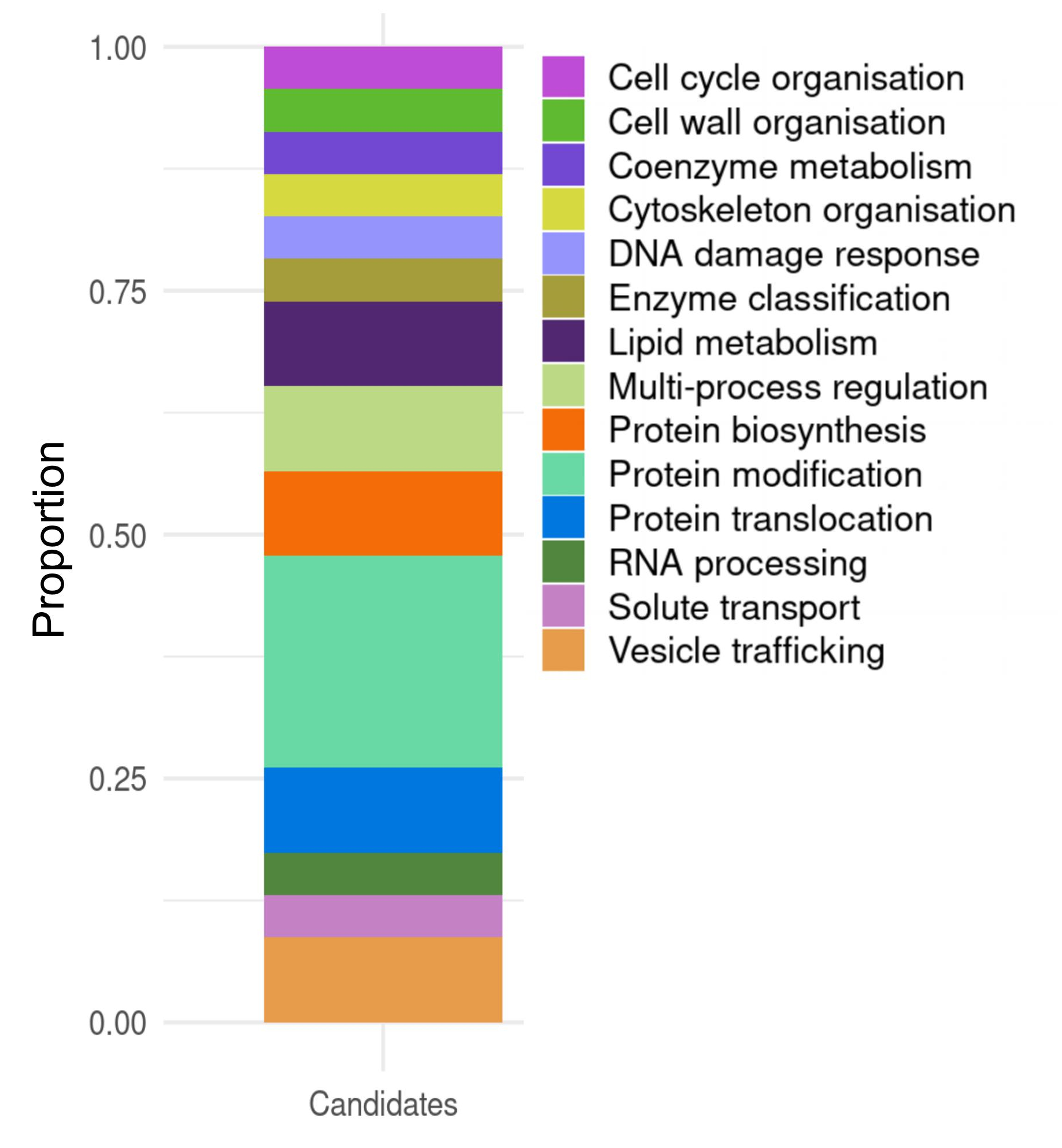


**Figure S11**. MapMan classification of the candidate genes. Colours indicate the main category, meanwhile, a more specific annotation is pinpointed at the right of the categories.


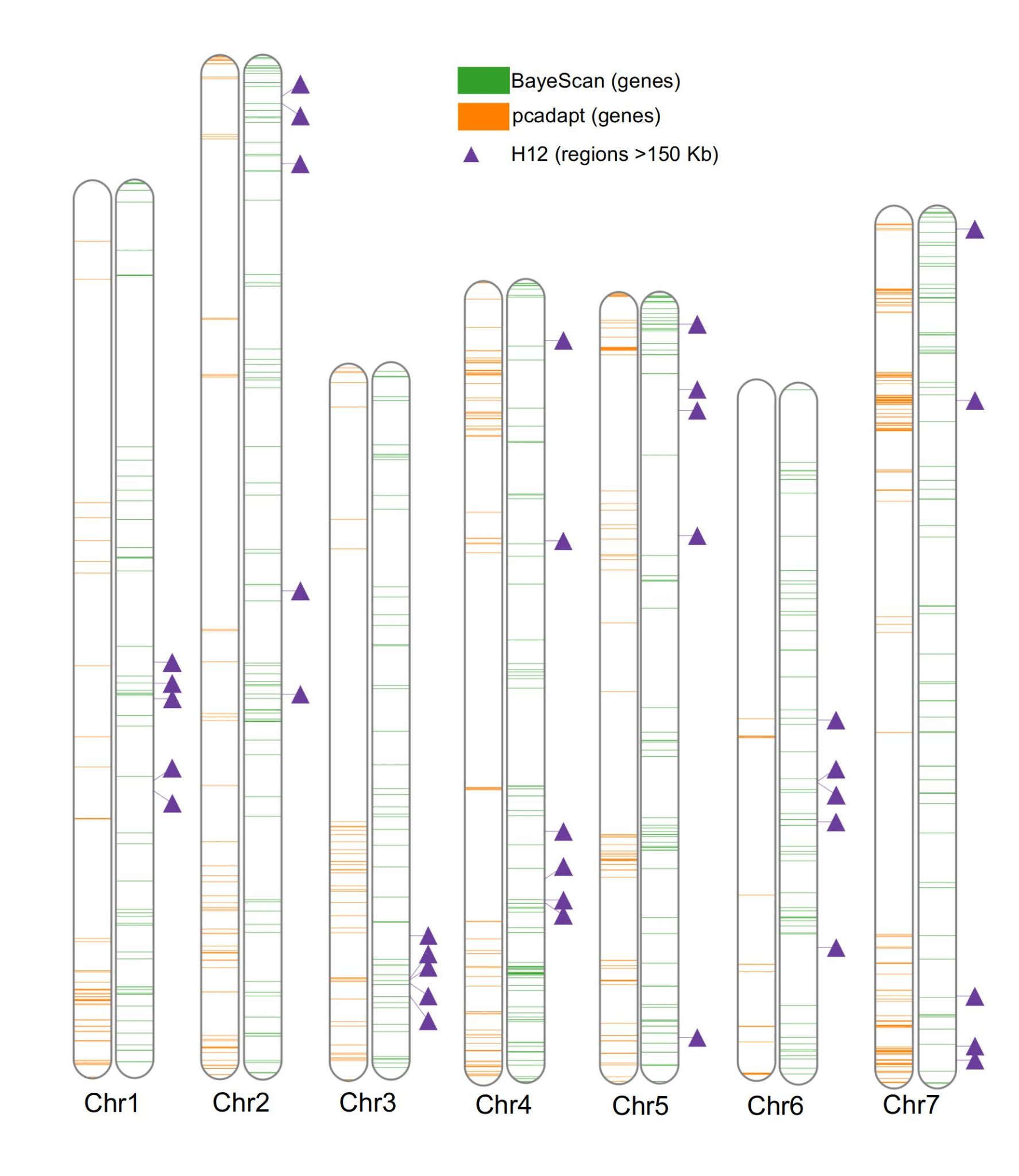


**Figure S12**. Genes where candidate SNPs were identified using BayeScan (green) and pcadapt (orange). Purple triangles show the top five selective sweeps found by H12.

**Table S2**. Candidates genes annotation.

| **Gene ID** | **MapMan category and subcategories** | **Description** |
| --- | --- | --- |
| Lcu.2rby.1G035900 (two hits) | Protein modification/phosphorylation/TKL protein kinase superfamily/G-Lectin protein kinase families  Protein modification/phosphorylation/TKL protein kinase superfamily | Protein kinase (SD-1)  Protein kinase (DUF26) |
|  |  |  |
| Lcu.2rby.1G066240 | Cell cycle organisation/mitosis and meiosis/sister chromatid separation/cohesin regulator complex/component SMC1 | Component SMC1/TTN8 of cohesin regulator complex. Structural maintenance of chromosomes protein |
| Lcu.2rby.1G074630  (two hits) | DNA damage response/DNA damage sensing and signalling/ | DNA single-strand break response kinase (ATR) |
|  | Protein modification/phosphorylation/atypical protein kinase families | Protein kinase (PIKK) |
| Lcu.2rby.2G010960 | not assigned/annotated | Uncharacterized GPI-anchored protein At1g61900 |
| Lcu.2rby.2G059530 | not assigned/annotated | DEAD-box ATP-dependent RNA helicase 46 |
| Lcu.2rby.3G061130 | not assigned/annotated | Pentatricopeptide repeat-containing protein At5g18475 |
| Lcu.2rby.3G061260 | Enzyme classification/EC_1 oxidoreductases/EC_1 | Pyridoxine/pyridoxamine 5-phosphate oxidase 1, chloroplastic & Enzyme classification.EC_1 oxidoreductases.EC_1.4 oxidoreductase acting on CH-NH2 group of donor (50.1.4 : 238.6) |
| Lcu.2rby.3G061270 | Protein biosynthesis/aminoacyl-tRNA synthetase activities | Proline-tRNA ligase |
| Lcu.2rby.3G061880 | Protein translocation/chloroplast/inner envelope TIC translocation system | Component Tic22 of inner envelope TIC translocation system |
| Lcu.2rby.3G071680 | Protein biosynthesis/translation elongation/eEF2 mRNA-translocation factor activity/regulatory eEF2 diphthamide-modification | Diphthine methyl ester synthase (DPH5) |
| Lcu.2rby.4G024020 | Vesicle trafficking/target membrane tethering/TRAPP (Trafficking-Protein-Particle) complexes/core components/component TRAPPC1 | Component BET5 of TRAPP-I/II/III complex-shared components |
| Lcu.2rby.4G043190 | RNA processing/pre-mRNA splicing/U2-type-intron-specific major spliceosome/U1 small nuclear ribonucleoprotein particle (snRNP)/accessory protein activities | Pre-mRNA-processing protein (PRP40C) |
| Lcu.2rby.4G066150 | Multi-process regulation/phosphoinositide lipid regulatory system/phosphatidylinositol 4/5-phosphate | Phosphatidylinositol 4-kinase (PI4K-beta) |
| Lcu.2rby.5G014340 | Vesicle trafficking/endomembrane trafficking/ESCRT (Endosomal Sorting Complex Required for Transport) complexes/ESCRT-II complex | Component VPS25 of ESCRT-II complex |
| Lcu.2rby.5G045180 | Protein modification/phosphorylation/TKL protein kinase superfamily/G-Lectin protein kinase families | Protein kinase (SD-1) |
| Lcu.2rby.5G046320 | not assigned | Anaphase-promoting complex subunit 1 |
| Lcu.2rby.6G025940 | Solute transport/carrier-mediated transport | Solute transporter (MTCC) |
| Lcu.2rby.7G006750 | Cell wall organisation/sporopollenin/biosynthesis | Long-chain fatty acid hydroxylase |
| Lcu.2rby.7G021070 | Coenzyme metabolism/tetrapyrrol biosynthesis/chlorophyll metabolism/chlorophyll(ide) interconversions/chlorophyll b reductase complex | Component NOL of chlorophyll b reductase complex |
| Lcu.2rby.7G021120 | not assigned/not annotated |  |
| Lcu.2rby.7G021130 | Multi-process regulation/Rop-GTPase regulatory system/RopGEF guanine nucleotide exchange factor activities | DHR2-type RopGEF guanine nucleotide exchange factor |
| Lcu.2rby.7G021140 | Protein translocation/chloroplast/inner envelope TIC translocation system/AAA-ATPase motor complex/FtsH12-FtsHi subcomplex/component FtsH12 | Component FtsH12 of protein translocation ATPase motor complex |
| Lcu.2rby.7G021150 | not assigned |  |
| Lcu.2rby.7G021160 | Lipid metabolism/lipid degradation/fatty acid degradation/auxiliary degradation activities | Dienoyl-CoA reductase |
| Lcu.2rby.7G021190 | Cytoskeleton organisation/microfilament network/myosin microfilament-based motor protein activities | Class VIII myosin microfilament-based motor protein |
| Lcu.2rby.7G021200 | not assigned/not annotated |  |
| Lcu.2rby.7G042060 | not assigned/annotated | Aldehyde dehydrogenase family 3 member F1 |
| Lcu.2rby.7G070800 | Lipid metabolism/fatty acid biosynthesis/acetyl-CoA generation/plastidial pyruvate dehydrogenase complex | Dihydrolipoamide dehydrogenase component E3 of plastidial pyruvate dehydrogenase complex |
| Lcu.2rby.7G070820 | Protein modification/phosphorylation/serine/threonine protein phosphatase superfamily/PPP Fe-Zn-dependent phosphatase families/PP4-class phosphatase complex | Regulatory component PP4R3 of PP4 phosphatase complex |
| Lcu.2rby.7G070850 | not assigned/annotated | Outer envelope pore protein 16-2, chloroplastic |
| Lcu.2rby.7G070870 | not assigned/not annotated |  |
| Lcu.2rby.7G070930 | not assigned/not annotated |  |
| Lcu.2rby.7G076470 | not assigned/annotated | Formin-like protein 13 |
